# Synthesis and spectroscopic properties of β-triazoloporphyrin–xanthone dyads

**DOI:** 10.3762/bjoc.11.155

**Published:** 2015-08-17

**Authors:** Dileep Kumar Singh, Mahendra Nath

**Affiliations:** 1 Department of Chemistry, University of Delhi, Delhi 110 007, India

**Keywords:** 1,3-dipolar cycloaddition, fluorescence, synthesis, triazoloporphyrin-xanthone dyads, UV–vis spectroscopy

## Abstract

A novel series of β*-*triazoloporphyrin–xanthone conjugates and xanthone-bridged β*-*triazoloporphyrin dyads has been synthesized in moderate to good yields through Cu(I)-catalyzed Huisgen 1,3-dipolar cycloaddition reaction of copper(II) 2-azido-5,10,15,20-tetraphenylporphyrin or zinc(II) 2-azidomethyl-5,10,15,20-tetraphenylporphyrin with various alkyne derivatives of xanthones in DMF containing CuSO_4_ and ascorbic acid at 80 °C. Furthermore, these metalloporphyrins underwent demetalation under acidic conditions to afford the corresponding free-base porphyrins in good to excellent yields. After successful spectroscopic characterization, these porphyrins have been evaluated for their photophysical properties. The preliminary results revealed a bathochromic shift in the UV–vis and fluorescence spectra of these porphyrin–xanthone dyads.

## Introduction

In the past few decades, porphyrin macrocycles have emerged as a unique class of heterocyclic compounds and as most attractive building blocks for supramolecular arrays. These molecules provide various desirable properties such as a rigid planar geometry, highly conjugated structures, intense electronic absorption and emission properties and small HOMO–LUMO energy gaps [1–2]. Additionally, the electronic properties of porphyrins can be modulated by introducing diverse functionalities on their periphery or changing the metal ions in the porphyrin core [3–4]. Due to the high thermal stability and extended π-electron system, porphyrins are very useful for the construction of molecular switches [5–6] and other organic photoelectric materials [7–8]. In addition, porphyrins are potentially used as photosensitizers in photodynamic therapy to treat various types of tumors [9–10]. In recent years, many hybrid molecules including porphyrin–C_60_ [11], porphyrin–quinones [12] and porphyrin–cyclodextrin [13] conjugates were synthesized and evaluated for their photophysical properties. These compounds have successfully demonstrated the occurrence of an excited electron transfer from the porphyrin subunit to the attached acceptor moieties. On the other hand, some photoinduced electron transfer systems such as porphyrin–tetrathiafulvalenes [14], porphyrin–cyanines [15], porphyrin–carotenes [16], porphyrin–arene diimide [17] and porphyrin–fluorocene or rhodamine [18] have also been synthesized in which the porphyrin unit acts as an electron acceptor.

Recently, the 1,2,3-triazole scaffold has been successfully employed to connect porphyrins with diverse functionalities such as quinolone [19], ferrocene [20], carbohydrate [21] and fullerene [22] through a copper(I)-catalyzed Huisgen–Sharpless–Meldal 1,3-dipolar cycloaddition reaction [23–24]. Some of these triazolo-bridged porphyrin dyads have shown an efficient intramolecular energy transfer between the porphyrin part and the attached subunit. Moreover, the 1,4-disubstituted triazoles are found to be very useful for various applications including modification of cell surfaces [25], synthesis of new glycoproteins [26], specific labeling of virus particles [27] and synthesis of diporphyrin analogues [20,28–30].

Xanthene-9*H*-ones are an important class of oxygen-containing heterocycles and are mainly found as secondary metabolites in higher plants and microorganisms. The naturally occurring and synthetic xanthones possess diverse pharmacological activities [31–34] including anti-oxidative [35], antihypertensive [36], anti-inflammatory [37] and antiplatelet agents [38]. They have also been used as fluorophores and exhibited good fluorescence properties when attached to a triazole ring [39–40]. Owing to the biological significance of porphyrins, 1,2,3-triazoles and xanthones, it was contemplated to incorporate these heterocyclic scaffolds in a single molecular framework to construct novel β-triazolo–porphyrin–xanthone conjugates and their diporphyrin analogues which may prove useful as photosensitizers for photodynamic therapy applications.

## Results and Discussion

In continuation of our interest to develop new synthetic methods for porphyrin analogues [41–46], we focus our attention on the construction of various novel β-substituted triazoloporphyrin–xanthone conjugates. For the preparation of these molecules, the alkynes, 3-amino-6-ethynylxanthen-9-one (**3**), 3-ethynyl-6-nitroxanthen-9-one (**4**), and 3-ethynyl-6-methoxyxanthen-9-one (**5**) were synthesized by using the literature procedures [39–40,47–49]. In addition, copper(II) 2-azido-5,10,15,20-tetraphenylporphyrin (**1**) [50] was synthesized in good yield after the treatment of copper(II) 2-amino-5,10,15,20-tetraphenylporphyrin with NaNO_2_ in THF in the presence of H_2_SO_4_ followed by the reaction with NaN_3_ [28]. The corresponding zinc(II) 2-azidomethyl-5,10,15,20-tetraphenylporphyrin (**2**) was prepared in 85% yield from zinc(II) 2-hydroxymethyl-5,10,15,20-tetraphenylporphyrin after the reaction with NaN_3_ and BF_3_·Et_2_O in 1,4-dioxane at 80 °C for 2 hours [51].

**Scheme 1:**
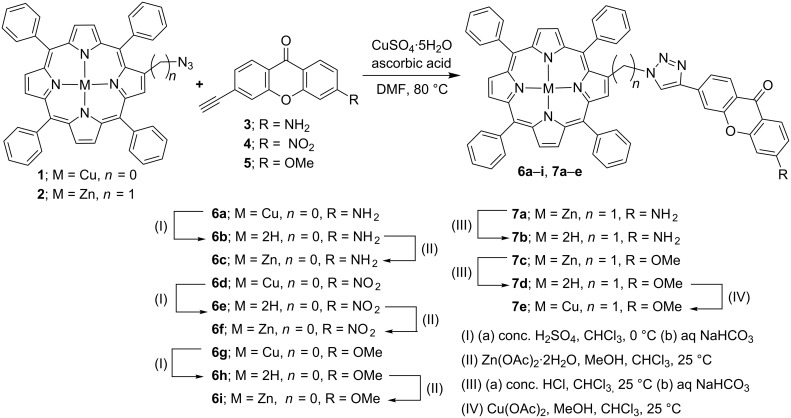
Synthesis of β-substituted triazoloporphyrin–xanthone conjugates **6a**–**i** and **7a**–**e**.

Initially, the copper(II) and zinc(II) derivatives of β-triazoloporphyrin–xanthone conjugates **6a**,**d**,**g** and **7a**,**c** were synthesized in 60–76% yields through a copper(I)-catalyzed Huisgen 1,3-dipolar cycloaddition reaction between copper(II) 2-azido-5,10,15,20-tetraphenylporphyrin (**1**) or zinc(II) 2-azidomethyl-5,10,15,20-tetraphenylporphyrin (**2**) with ethynyl-substituted xanthones **3**, **4** or **5**, using copper sulfate and ascorbic acid in DMF at 80 °C (Scheme 1). Further, the demetalation of the copper(II) porphyrins **6a,d**,**g** with conc. H_2_SO_4_ at 0 °C and zinc(II) derivatives **7a**,**c** with conc. HCl at 25 °C and neutralization with 5% aq NaHCO_3_ afforded the corresponding free-base porphyrins **6b**,**e**,**h** and **7b**,**d**, respectively in 78–83% yields. In addition, the zinc porphyrins **6c**,**f**,**i** and copper porphyrin **7e** were prepared in 90–93% yields from free-base porphyrins **6b**,**e**,**h** and **7d** by their treatment with zinc acetate and copper acetate respectively, in a CHCl_3_–MeOH mixture at room temperature (Scheme 1).

**Scheme 2:**
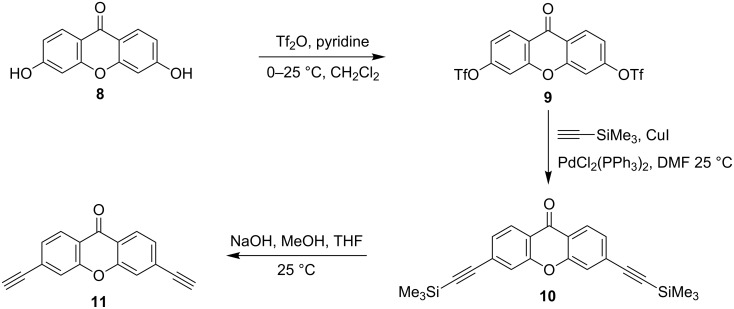
Synthesis of 3,6-diethynyl-xanthen-9-one (**11**).

Further, this methodology was extended to the preparation of symmetrical xanthone-bridged triazolodiporphyrins (**12a**,**b** and **13a**–**c**). For the synthesis of these compounds, 3,6-diethynylxanthen-9-one (**11**) was synthesized in three steps from 3,6-dihydroxyxanthen-9-one (**8**) as a starting material. The first step involved the reaction of xanthone (**8**) with triflic anhydride in CH_2_Cl_2_ containing pyridine at 0 °C to afford 3,6-di-OTf-xanthone [52] (**9**). Subsequent Sonogashira coupling with trimethylsilylacetylene followed by the deprotection of the trimethylsilyl group in the presence of aqueous NaOH at room temperature produced the desired product 3,6-diethynylxanthen-9-one (**11**) in 81% yield (Scheme 2) [39].

**Scheme 3:**
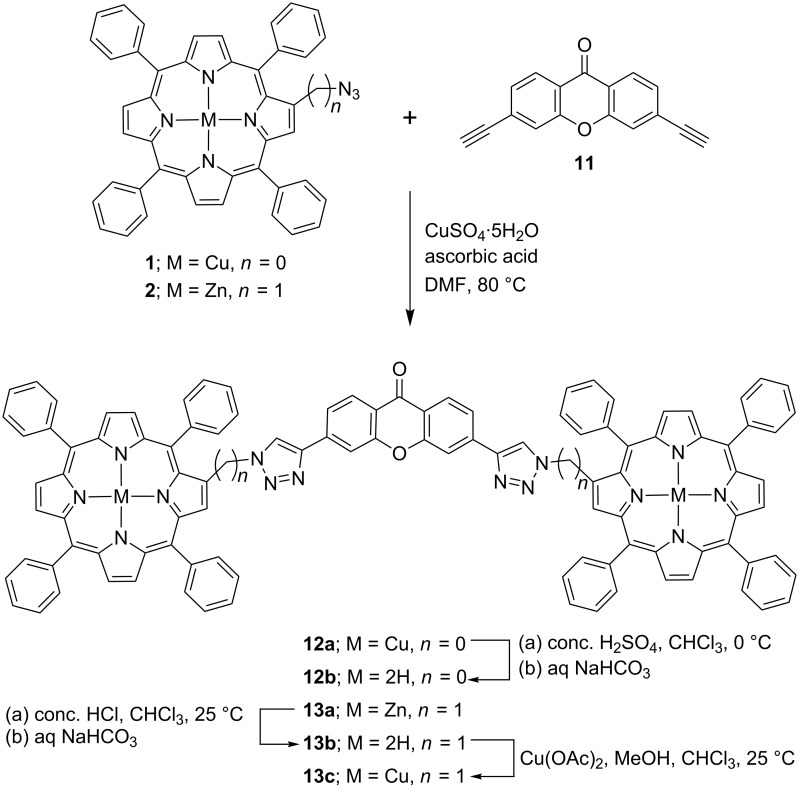
Synthesis of xanthone-bridged triazolo-bisporphyrins **12a**,**b** and **13a**–**c**.

Finally, the copper and zinc derivatives of the xanthone-bridged triazolodiporphyrins (**12a** and **13a**) were synthesized in good yields by the “click reaction” of 3,6-diethynylxanthen-9-one (**11**) with copper and zinc azidoporphyrins, respectively. Further, the free-base diporphyrins **12b** and **13b** were obtained in 81% and 84% yields, respectively after the treatment of **12a** with conc. H_2_SO_4_ at 0 °C and **13a** with conc. HCl at 25 °C in chloroform. In addition, the copper(II) bisporphyrin **13c** was obtained in almost quantitative yield by reacting the free-base porphyrin **13b** with Cu(OAc)_2_ in a CHCl_3_–MeOH mixture at room temperature (Scheme 3).

All the newly synthesized products were well purified by column chromatography and characterized by NMR, IR, UV–vis and mass spectral data in addition to elemental analyses. The ^1^H NMR spectrum of xanthone **10** showed a characteristic singlet of 18 protons at δ 0.28 ppm due to trimethylsilyl group which revealed the formation of compound **10**. A characteristic singlet of alkyne protons in xanthones **4** and **11** were found at δ 4.69 ppm and δ 4.66 ppm, respectively. In addition, the IR spectra of all the three xanthones **4**, **10** and **11** showed a C≡C-bond stretching at around 2100–2157 cm^−1^ which confirms the attachment of the alkyne moiety to the xanthone core.

In the ^1^H NMR spectra, 7 β-pyrrolic protons of porphyrin–xanthone conjugates (**6b**,**c**,**e**,**f**,**h**,**i** and **7a**–**d**) and 14 β-pyrrolic protons of bisporphyrins (**12b** and **13a**,**b**) were found in the down-field region between δ 8.44–9.07 ppm as either singlet, doublet or multiplet. The 20 *meso*-phenyl protons of compounds **6b**,**c**,**e**,**f**,**h**,**i** and **7a**–**d** and the 40 *meso*-phenyl protons of bisporphyrins **12b** and **13a**,**b** were assigned to doublets and multiplets in the region between δ 6.99–8.26 ppm. In addition, 6 xanthone protons in the case of all porphyrin–xanthone dyads were found between δ 6.47–8.41 ppm as doublet, double doublet or multiplet. A characteristic singlet of two protons at around δ −2.7 ppm was assigned for internal NH protons of the porphyrin core in the case of all the free-base porphyrins. In porphyrins **7a**–**d** and **13a**,**b**, a singlet for the methylene protons was observed between δ 5.76–5.82 ppm. Similarly, a singlet of three protons due to the presence of methoxy group was observed in the upfield region at δ ~3.9 ppm in porphyrins **6h**,**i** and **7c**,**d**. The two protons for the amino group in porphyrins **6b** and **6c** appeared as broad singlet at δ 4.33 and 4.89 ppm, respectively, whereas these protons are down-field shifted to δ ~6.52 ppm in the case of porphyrins **7a** and **7b**. The IR spectra of the compounds containing amino groups showed a NH_2_ bond stretching band between 3348–3433 cm^−1^, whereas all the free-base porphyrins showed absorptions between 3322–3327 cm^−1^ due to the internal NH groups of the porphyrin core. In addition, a strong band was observed at 1602–1654 cm^−1^ due to the presence of the carbonyl groups in the porphyrin–xanthone dyads. The mass spectra of all compounds further supported the assigned structures by showing a molecular ion peak either as [M + H]^+^ or [M + Na]^+^ or [M + K]^+^.

**Figure 1:**
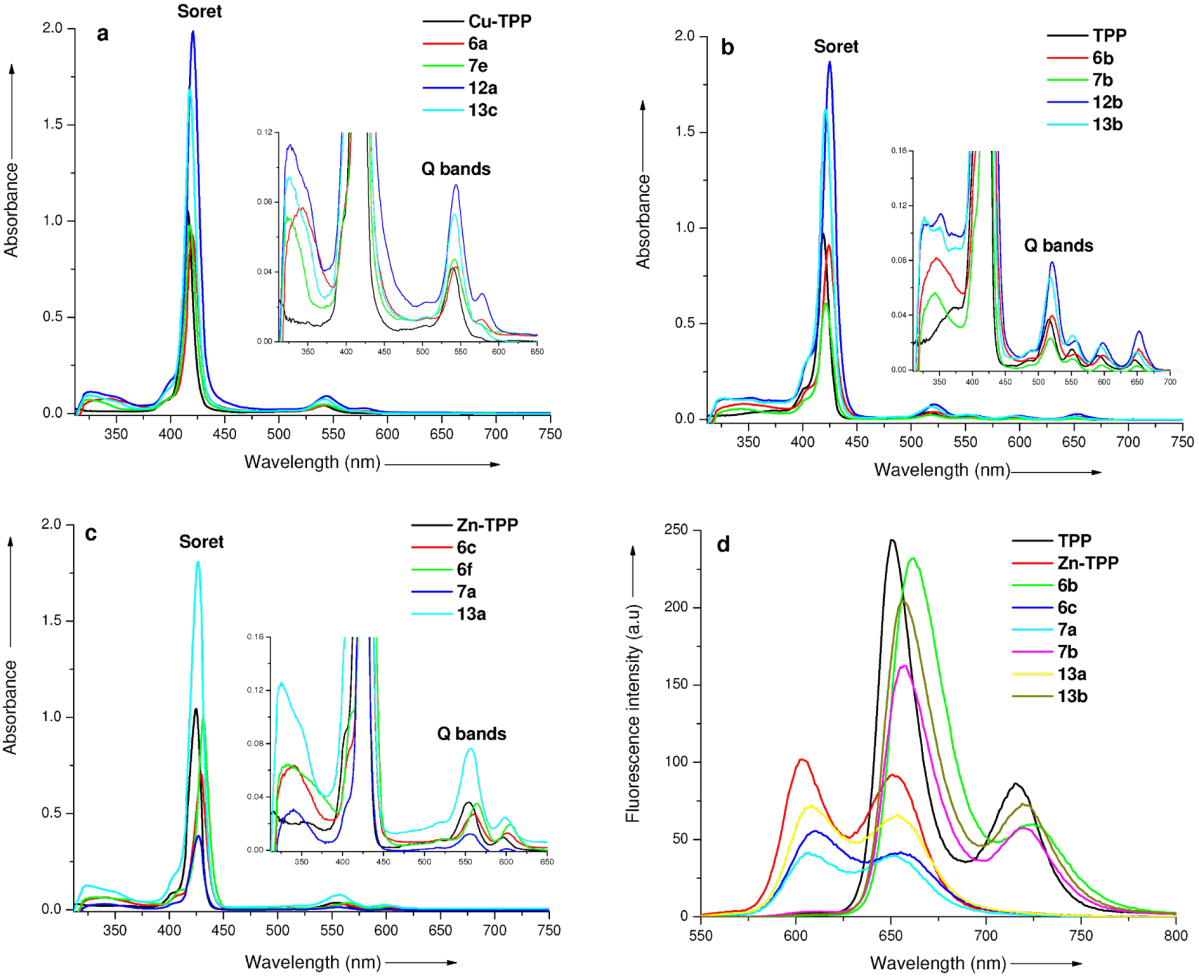
(a) Electronic absorption spectra of Cu-TPP, **6a**, **7e**, **12a** and **13c**. (b) Electronic absorption spectra of TPP, **6b**, **7b**, **12b** and **13b**. (c) Electronic absorption spectra of Zn-TPP, **6c**, **6f**, **7a** and **13a** in CHCl_3_ (1.5 × 10^−6^ mol L^−1^) at 298 K and inset shows the expanded UV–vis spectra with Q-bands. (d) Fluorescence spectra of porphyrins TPP, Zn-TPP, **6b**,**c**, **7a**,**b**, and **13a**,**b** in CHCl_3_ (1.5 × 10^−6^ mol L^−1^) at 298 K, λ_ex_ = 420 nm.

The electronic absorption spectra of all the newly prepared porphyrin–xanthone conjugates were taken in CHCl_3_ (1.5 × 10^−6^ M) at room temperature. The copper(II) β-triazoloporphyrin–xanthone conjugates **6a**,**d**,**g** and **12a** exhibited a typical Soret band at around 420–421 nm and two Q-bands at ~544 and 578 nm, which were found to be red-shifted by about 3–5 nm as compared to the starting porphyrin Cu-TPP (Soret band at 416 nm and Q-bands at 541 and 574 nm). Further, the free-base β-triazoloporphyrin–xanthone conjugates **6b**,**e**,**h** and **12b** display their Soret bands at around 424–425 nm and four Q-bands around ~521, 556, 596 and 652 nm which were also red-shifted by about 5–6 nm when compared to the TPP (Soret band at 419 nm and Q-bands at 516, 551, 590 and 646 nm). In addition, zinc(II) β-triazoloporphyrin–xanthone dyads **6c**,**f**,**i** exhibited their Soret bands between 430–431 nm and two Q-bands at ~561 and 602 nm which were found to be red-shifted by about 5–6 nm as compared to the Zn-TPP (Soret band at 425 nm and Q-bands at 555 and 597 nm). In contrast, zinc(II) β-triazolomethylporphyrin–xanthone dyads (**7a**,**c**,**13a**), free-base β-triazolomethylporphyrin–xanthone dyads (**7b**,**d**,**13b**) and copper(II) β-triazolomethylporphyrin–xanthone dyads (**7e**,**13c**) exhibited only a 2 nm red shift in their Soret and Q-bands when compared with the Zn-TPP, TPP and Cu-TPP, respectively. Thus, the electronic absorption spectra of β-triazoloporphyrin–xanthone conjugates exhibited 2–4 nm red shifts in their Soret and Q-bands as compared to the β-triazolomethylporphyrin–xanthone conjugates. In addition, the absorption intensity of symmetrical xanthone-bridged β*-*triazolodiporphyrins (**12a**,**b** and **13a**–**c**) was quite larger than their corresponding monoporphyrin–xanthone conjugates. The electronic absorption spectra of copper porphyrins (Cu-TPP, **6a**, **7e**, **12a** and **13c**), free-base porphyrins (TPP, **6b**, **7b**, **12b** and **13b**), and zinc porphyrins (Zn-TPP, **6c**, **6f**, **7a** and **13a**) are shown in Figure 1a–c.

Besides the Soret and Q-bands, an additional absorption band was also observed at ~340 nm in the UV–vis spectra of these porphyrin–xanthone conjugates due to the presence of xanthone moiety (Figure 1a–c) which suggests that there is no significant interaction between the xanthone and porphyrin moieties in the ground state [53–55].

The fluorescence spectra of compounds **6b**,**c**, **7a**,**b**, and **13a**,**b** depicted in Figure 1d showed two emission bands of free-base porphyrin–xanthone conjugates between 656–725 nm which are slightly quenched, but red-shifted about 5–10 nm as compared to the TPP (emission bands at 650 and 715 nm). Similarly, the two emission bands of zinc porphyrins were also observed between 607–662 nm and they were also quenched and red-shifted by about 5–10 nm as compared to the Zn-TPP (emission bands at 602 and 651 nm). In contrast, the copper porphyrins did not show any significant emission due to the paramagnetic nature of the Cu(II) ions [56].

## Conclusion

In summary, we have successfully synthesized and characterized two new alkyne-substituted xanthones, 3-ethynyl-6-nitroxanthen-9-one and 3,6-diethynylxanthen-9-one. In addition, a new series of various β-triazole-linked porphyrin–xanthone conjugates and xanthone-bridged triazoloporphyrin dyads were synthesized through click chemistry in moderate to good yields. The preliminary photophysical evaluation of these π-conjugated molecules revealed a bathochromic shift in their electronic absorption and fluorescence spectra as compared to the *meso*-tetraarylporphyrins. These results are significantly encouraging and henceforth may be useful for the development of new porphyrin materials for various applications including photosensitizers for photodynamic applications.

## Supporting Information

File 1 Experimental details and characterization data.

File 2 ^1^H and ^13^C NMR spectra of newly synthesized compounds.
