# Supplementary material for: Synthesis and spectroscopic properties of β-triazoloporphyrin–xanthone dyads
Source: Beilstein J Org Chem. 2015 Aug 17;11:1434–40. doi: 10.3762/bjoc.11.155 (PMC4578393; doi:10.3762/bjoc.11.155)
Supplement: File 1 — Experimental details and characterization data. [file Beilstein_J_Org_Chem-11-1434-s001.pdf]

Supporting Information File 1

for

Synthesis and spectroscopic properties of β-triazoloporphyrin–xanthone dyads

Dileep Kumar Singh and Mahendra Nath*

Address: Department of Chemistry, University of Delhi, Delhi 110 007, India

Email: Mahendra Nath* - mnath@chemistry.du.ac.in

*Corresponding author

Experimental details and characterization data

General:

All chemicals and solvents were purchased either from Sigma–Aldrich or Merck. Thin layer chromatography (TLC) was performed using silica gel 60 F_{254} (pre-coated aluminium sheets) from Merck. Reactions were monitored by TLC and products were purified by column chromatography using silica gel 60–120 mesh or activated neutral aluminum oxide (Brokmann grade I-II, Merck). ^{1}H NMR (400 MHz) and ^{13}C NMR (100 MHz) spectra were recorded in $CDCl_3$ (δ = 7.26 ppm for ^{1}H NMR and δ = 77.00 ppm for ^{13}C NMR) or DMSO-d_6 (δ = 2.50 ppm for ^{1}H NMR and δ = 39.50 ppm for ^{13}C

NMR), using a Jeol ECX 400P (400 MHz) NMR spectrometer and chemical shifts were expressed in parts per million (ppm). The coupling constants were denoted as (*J*) and are reported in Hertz (Hz). Elemental analyses for newly prepared compounds were performed on an Elementar Analysensysteme GmbH VarioEL elemental analyzer. Infrared spectra were recorded on a Perkin Elmer IR spectrometer as film ($CHCl_3$) or KBr pellets and absorption maxima (ν_{max}) are given in cm^{-1}. The ESI mass spectra were recorded on a THERMO Finnigan LCQ Advantage max ion trap mass spectrometer or on an Agilent 6530AA QTOF LCMS in positive mode. UV–vis spectra were recorded on an Analytic jena Specord 250 UV–vis spectrophotometer using $CHCl_3$ as a solvent. Fluorescence spectra were recorded on a Varian Cary Eclipse fluorescence spectrophotometer. Melting points of the products were recorded in open capillary tubes using a Büchi M-560 melting point apparatus and are uncorrected. 3-Amino-6-ethynyl-xanthen-9-one (**3**), 3-ethynyl-6-nitro-xanthen-9-one (**4**), and 3-ethynyl-6-methoxyxanthen-9-one (**5**) were synthesized following literature procedures [1-5]. The alkynes **3** and **5** are known and their spectral data were in agreement with the reported data while the characterization data of unknown alkyne **4** are given below.

3-Ethynyl-6-nitro-xanthen-9-one (4). Yellow solid, yield: 78%; mp 148-150 °C; IR (KBr) ν_{max}: 2919, 2850, 2106, 1614, 1441, 1418, 1275, 1256, 1168, 1104, 1022, 843, 772, 666 cm^{-1}; ^1H NMR (400 MHz, DMSO-d_6) δ_H: 8.43 (d, J = 2.20 Hz, 1H), 8.37 (d, J = 8.79 Hz, 1H), 8.20 (dd, J_1 = 8.79 Hz and J_2 = 2.20 Hz, 1H), 8.16 (d, J = 8.05 Hz, 1H), 7.80 (d, J = 1.46 Hz, 1H), 7.55 (dd, J_1 = 8.05 Hz and J_2 = 1.46 Hz, 1H), 4.69 (s, 1H) ppm; ESI-MS: m/z = 266.2 $(M+H)^+$; Anal. Calcd for $C_{15}H_7NO_4$: C, 67.93; H, 2.66; N, 5.28; found: C, 68.05; H, 2.73; N, 5.19.

Synthesis of 3,6-bis(trimethylsilanylethynyl)-xanthen-9-one (10)

A mixture of 3,6-bis(trifluoromethanesulfonyl)xanthone [6] (**9**; 490 mg, 1.0 mmol), $PdCl_2(PPh_3)_2$ (140.4 mg, 0.2 mmol) and CuI (76.2 mg, 0.4 mmol) in dry DMF was degassed by bubbling with nitrogen gas for 30 min. Triethylamine (2.78 mL, 20 mmol) was added followed by trimethylsilylacetylene (0.56 mL, 4 mmol). The reaction mixture was stirred for 2 h at rt. After completion of the reaction, the reaction mixture was poured into water (40 mL) and extracted with ethyl acetate (20 mL × 3). The organic layers were combined and washed with brine, dried over Na_2SO_4 and concentrated under reduced pressure. The crude product was purified by column chromatography over silica gel using 5% EtOAc in hexane as eluent to give the product as white solid with 81% yield. IR (KBr) ν_{max}: 2960, 2157, 1666, 1610, 1423, 1251, 1169, 988, 842, 760, 701 cm^{-1}; ^1H NMR (400 MHz, $CDCl_3$) δ_H: 8.22 (d, J = 8.05 Hz, 2H), 7.56 (s, 2H), 8.05 (dd, J_1 = 8.05 Hz and J_2 = 1.46 Hz, 2H), 0.28 (s, 18H) ppm; ESI-HRMS: m/z = 389.1388 $(M+H)^+$.

Synthesis of 3,6-diethynylxanthen-9-one (11)

To a solution of 3,6-bis(trimethylsilanylethynyl)xanthen-9-one (**10**; 387 mg, 1.0 mmol) in 33% MeOH in THF (30 mL) was added 1 N aqueous NaOH (10 mL) at rt and the reaction mixture was stirred for 30 min. After completion of the reaction, the mixture was extracted with EtOAc (20 mL × 2). The organic layers were combined, washed with brine, dried over Na_2SO_4 and concentrated in vacuo. The obtained residue was purified by column chromatography over silica gel using 5% EtOAc in hexane as the eluent to give the product as pale yellow solid with 81% yield. mp >300 °C; IR (KBr) ν_{max}: 2923, 2853, 2100, 1663, 1612, 1423, 1119, 866, 778, 646 cm^{-1}; ^1H NMR (400 MHz, DMSO-d_6) δ_H: 8.15 (d, J = 8.05 Hz, 2H), 7.76 (d, J = 1.46 Hz, 2H), 7.53 (dd, J_1

= 8.05 Hz, J_2 = 1.46 Hz, 2H), 4.66 (s, 2H) ppm; ^{13}C NMR (100 MHz, DMSO-d$_6$) δ_C: 175.10, 155.38, 128.65, 127.72, 126.59, 121.34, 121.28, 85.47, 82.10 ppm; ESI-MS: m/z = 245 (M+H)$^+$; Anal. Calcd for $C_{17}H_8O_2$: C, 83.60; H, 3.30; found: C, 83.34; H, 3.32.

General procedure for the synthesis of copper(II) β-triazoloporphyrin–xanthone dyads (6a, 6d, 6g) and copper(II) xanthone-bridged β-triazolodiporphyrin 12a

To a solution of copper(II) 2-azido-5,10,15,20-tetraphenylporphyrin (100 mg, 0.139 mmol) in DMF (10 mL), $CuSO_4{\cdot}5H_2O$ (6.94 mg, 0.028 mmol), ascorbic acid (9.79 mg, 0.055 mmol) and ethynyl-substituted xanthones (0.166 mmol for the synthesis of compounds **6a**, **6d**, **6g** and 0.083 mmol for the formation of compound **12a**) were added and the reaction mixture was stirred at 80 °C for 2–4 h under a nitrogen atmosphere in the dark. The progress of the reaction was monitored by thin layer chromatography (TLC). After completion of the reaction, the mixture was allowed to cool to room temperature and 50 mL chloroform were added. The resulting solution was washed thoroughly with water, the organic layer was dried over anhydrous sodium sulfate and the solvent was evaporated under reduced pressure. The crude product was purified by column chromatography over neutral alumina using chloroform as the eluent.

Copper(II) 3-amino-6-[1-(5,10,15,20-tetraphenylporphyrin-2-yl)-1*H*-[1,2,3]triazol-4-yl]-xanthen-9-one (6a). Purple solid; yield: 61%; mp >300 °C; UV λ_{max} ($\varepsilon\times10^{-4}$, M^{-1} cm^{-1}): 420 (93.04), 544 (4.30), 578 (1.28) nm; IR ($CHCl_3$) ν_{max}: 3348, 1610, 1438, 1344, 1074, 1004, 799, 753, 702 cm^{-1}; ESI-MS: m/z = 952.0 (M+H)$^+$; Anal. Calcd for $C_{59}H_{36}CuN_8O_2{\cdot}H_2O$: C, 73.01; H, 3.95; N, 11.55; found: C, 72.89; H, 4.12; N, 11.42.

Copper(II) 3-nitro-6-[1-(5,10,15,20-tetraphenylporphyrin-2-yl)-1*H*-[1,2,3]triazol-4-yl]-xanthen-9-one (6d). Purple solid; yield: 67%; mp 263-265 °C; UV λ_{max} ($\varepsilon\times10^{-4}$, M^{-1} cm^{-1}): 420 (92.39), 544 (3.85), 578 (0.92) nm; IR ($CHCl_3$) ν_{max}: 3056, 2922, 2851, 1667, 1624, 1599, 1531, 1439, 1345, 1301, 1230, 1176, 1074, 1004, 880, 799, 754, 702 cm^{-1}; ESI-MS: m/z = 1020.2 $(M+K)^+$; Anal. Calcd for $C_{59}H_{34}CuN_8O_4$: C, 72.13; H, 3.49; N, 11.40; found: C, 71.98; H, 3.52; N, 11.54.

Copper(II) 3-methoxy-6-[1-(5,10,15,20-tetraphenylporphyrin-2-yl)-1*H*-[1,2,3]triazol-4-yl]-xanthen-9-one (6g). Purple solid; yield: 60%; mp 238-240 °C; UV λ_{max} ($\varepsilon\times10^{-4}$, M^{-1} cm^{-1}): 420 (83.01), 544 (3.49), 576 (0.83) nm; IR ($CHCl_3$) ν_{max}: 3056, 2919, 2850, 1612, 1437, 1344, 1270, 1254, 1205, 1158, 1074, 1027, 1004, 798, 753, 701 cm^{-1}; ESI-MS: m/z = 967.2 $(M+H)^+$; Anal. Calcd for $C_{60}H_{39}CuN_7O_3.2H_2O$: C, 71.81; H, 4.12; N, 9.77; found: C, 72.12; H, 4.16; N, 9.78.

Copper(II) 3,6-bis-[1-(5,10,15,20-tetraphenylporphyrin-2-yl)-1*H*-[1,2,3]triazol-4-yl]-xanthen-9-one (12a). Purple solid; yield: 43%; mp >300 °C; UV λ_{max} ($\varepsilon\times10^{-4}$, M^{-1} cm^{-1}): 421 (198.79), 544 (9.01), 578 (2.75) nm; IR ($CHCl_3$) : 3056, 1617, 1489, 1438, 1344, 1196, 1073, 1004, 934, 799, 753, 701 cm^{-1}; ESI-MS: m/z = 1677.3 $(M+H)^+$; Anal. Calcd for $C_{105}H_{62}Cu_2N_{14}O_2.2H_2O$: C, 73.54; H, 3.88; N, 11.44; found: C, 73.49; H, 4.11; N, 11.42.

General procedure for the synthesis of free-base β-triazoloporphyrin–xanthone dyads (6b, 6e, 6h) and xanthone-bridged β-triazolodiporphyrin 12b

The free-base porphyrins **6b**, **6e**, **6h** and **12b** were synthesized from the corresponding copper(II) porphyrin–xanthone dyads in a similar manner as described in the literature [7]. The crude products were purified by column chromatography on neutral alumina using 60–80% chloroform in hexane as eluent.

3-Amino-6-[1-(5,10,15,20-tetraphenylporphyrin-2-yl)-1*H*-[1,2,3]triazol-4-yl]-xanthen-9-one (6b). Purple solid; yield: 78%; mp >300 °C; UV λ_{max} ($\varepsilon \times 10^{-4}$, M^{-1} cm^{-1}): 424 (91.12), 521 (3.97), 556 (1.15), 596 (1.06), 652 (1.56) nm; λ_{Em}($CHCl_3$; λ_{Ex} 420 nm): 661, 725 nm; IR (KBr) ν_{max}: 3395, 2920, 1608, 1435, 1345, 1302, 1072, 1000, 964, 798, 718, 699 cm^{-1}; ^1H NMR (400 MHz, $CDCl_3$) δ_H: 9.05 (s, 1H, *β*-pyrrolic H), 8.96-8.93 (m, 2H, *β*-pyrrolic H), 8.85 (d, *J* = 4.88 Hz, 1H, *β*-pyrrolic H), 8.79-8.76 (m, 2H, *β*-pyrrolic H), 8.73 (d, *J* = 4.88 Hz, 1H, *β*-pyrrolic H), 8.36 (d, *J* = 7.93 Hz, 1H, ArH), 8.25-8.19 (m, 6H, *meso*-ArH), 8.15 (d, *J* = 8.54 Hz, 1H, ArH), 7.97 (d, *J* = 6.71 Hz, 2H, *meso*-ArH), 7.89 (s, 1H, triazole H), 7.80-7.73 (m, 10H, *meso*-ArH and ArH), 7.55 (d, *J* = 7.93 Hz, 1H, ArH), 7.37-7.33 (m, 2H, *meso*-ArH), 7.22-7.18 (m, 1H, *meso*-ArH), 6.65-6.62 (m, 2H, ArH), 4.33 (s, 2H, NH_2), -2.69 (s, 2H, internal NH) ppm; ^{13}C NMR (100 MHz, $CDCl_3$) δ_C: 175.51, 158.37, 156.36, 152.77, 145.80, 141.73, 141.60, 141.44, 139.14, 136.09, 134.56, 133.92, 129.73, 129.03, 128.85, 128.42, 128.17, 127.93, 127.84, 127.01, 126.86, 126.81, 126.78, 126.52, 125.10, 121.74, 121.27, 121.02, 120.76, 120.74, 119.38, 114.02, 113.74, 112.44, 99.83 ppm; ESI-MS: m/z = 891.3 $(M+H)^+$; Anal. Calcd for $C_{59}H_{38}N_8O_2$: C, 79.53; H, 4.30; N, 12.58; found: C, 79.81; H, 4.18; N, 12.36.

3-Nitro-6-[1-(5,10,15,20-tetraphenylporphyrin-2-yl)-1*H*-[1,2,3]triazol-4-yl]-xanthen-9-one (6e). Purple solid; yield: 81%; mp >300 °C; UV λ_{max} ($\varepsilon \times 10^{-4}$, M^{-1} cm^{-1}): 424 (92.94), 521 (4.01), 554 (1.15), 596 (1.04), 653 (1.48) nm; λ_{Em}($CHCl_3$; λ_{Ex} 420 nm): 661, 725 nm; IR ($CHCl_3$) ν_{max}: 3326, 3056, 2923, 1654, 1624, 1530, 1474, 1438, 1345, 1216, 1177, 1073, 1030, 965, 879, 800, 752, 701 cm^{-1}; ^1H NMR (400 MHz, $CDCl_3$): δ_H: 9.05 (s, 1H, *β*-pyrrolic H), 8.95 (s, 2H, *β*-pyrrolic H), 8.86-8.72 (m, 4H, *β*-pyrrolic H), 8.36-8.35 (m, 1H, ArH), 8.25-8.19 (m, 7H, *meso*-ArH and ArH), 7.98 (d, *J* = 6.59 Hz, 2H, *meso*-ArH), 7.93 (s, 1H, triazole H), 7.78-7.74 (m, 10H,

meso-ArH and ArH), 7.59-7.57 (m, 1H, ArH), 7.37-7.33 (m, 2H, *meso*-ArH), 7.22-7.20 (m, 1H, *meso*-ArH), 6.97-6.92 (m, 2H, ArH), -2.70 (s, 1H, internal NH) ppm; ^{13}C NMR (100 MHz, $CDCl_3$) δ_C: 175.33, 156.74, 155.55, 150.99, 145.16, 141.71, 141.59, 141.41, 139.32, 138.22, 134.57, 134.33, 133.99, 133.95, 128.40, 128.21, 127.97, 127.74, 127.46, 126.90, 126.84, 126.80, 126.53, 125.56, 125.48, 122.32, 121.79, 120.86, 120.80, 120.77, 119.17, 117.92, 114.30, 114.07 ppm; ESI-MS: m/z = 921.2 $(M+H)^+$; Anal. Calcd for $C_{59}H_{36}N_8O_4.2H_2O$: C, 74.05; H, 4.21; N, 11.71; found: C, 74.26; H, 4.28; N, 11.68.

3-Methoxy-6-[1-(5,10,15,20-tetraphenylporphyrin-2-yl)-1*H*-[1,2,3]triazol-4-yl]-xanthen-9-one (6h). Purple solid; yield: 79%; mp 278-280 °C; UV λ_{max} ($\varepsilon \times 10^{-4}$, M^{-1} cm^{-1}): 424 (75.34), 521 (3.07), 555 (0.68), 596 (0.56), 653 (1.01) nm; λ_{Em}($CHCl_3$; λ_{Ex} 420 nm): 661, 725 nm; IR ($CHCl_3$) ν_{max}: 3322, 3059, 2925, 1612, 1437, 1348, 1271, 1205, 1157, 1029, 981, 965, 800, 752, 701 cm^{-1}; ^{1}H NMR (400 MHz, $CDCl_3$) δ_H: 9.05 (s, 1H, *β*-pyrrolic H), 8.95 (s, 2H, *β*-pyrrolic H), 8.85 (d, *J* = 4.88 Hz, 1H, *β*-pyrrolic H), 8.79-8.76 (m, 2H, *β*-pyrrolic H), 8.73 (d, *J* = 4.88 Hz, 1H, *β*-pyrrolic H), 8.38 (d, *J* = 7.93 Hz, 1H, ArH), 8.28 (d, *J* = 8.54 Hz, 1H, ArH), 8.25-8.19 (m, 6H, *meso*-ArH), 7.98 (d, *J* = 6.71 Hz, 2H, *meso*-ArH), 7.94 (s, 1H, triazole H), 7.79-7.75 (m, 10H, *meso*-ArH and ArH), 7.59 (d, *J* = 7.93 Hz, 1H, ArH), 7.38-7.34 (m, 2H, *meso*-ArH), 7.22-7.18 (m, 1H, *meso*-ArH), 6.98-6.95 (m, 2H, ArH), 3.96 (s, 3H, $-OCH_3$), -2.69 (s, 2H, internal NH) ppm; ^{13}C NMR (100 MHz, $CDCl_3$) δ_C: 175.90, 165.05, 158.16, 156.57, 145.67, 141.74, 141.62, 141.45, 139.22, 136.58, 134.56, 133.95, 129.70, 129.00, 128.83, 128.20, 127.15, 126.88, 126.81, 126.78, 126.53, 125.20, 121.75, 121.34, 121.14, 120.80, 120.76, 119.33, 115.88, 114.12, 113.33, 100.19, 55.86 ppm; ESI-MS: m/z = 906.3 $(M+H)^+$; Anal. Calcd for $C_{60}H_{39}N_7O_3.H_2O$: C, 77.99; H, 4.47; N, 10.61; found: C, 78.14; H, 4.53; N, 10.51.

3,6-Bis[1-(5,10,15,20-tetraphenylporphyrin-2-yl)-1*H*-[1,2,3]triazol-4-yl]-xanthen-9-one (12b). Purple solid; yield: 81%; mp >300 °C; UV λ_{max} ($\varepsilon\times10^{-4}$, M^{-1} cm^{-1}): 425 (186.96), 521 (7.89), 555 (2.11), 597 (1.98), 653 (2.85) nm; λ_{Em}($CHCl_3$; λ_{Ex} 420 nm): 663, 727 nm; IR ($CHCl_3$) ν_{max}: 3327, 2921, 1617, 1474, 1438, 1346, 1178, 1073, 1001, 1029, 981, 965, 934, 800, 752, 700 cm^{-1}; ^{1}H NMR (400 MHz, $CDCl_3$) δ_H: 9.07 (s, 2H, *β*-pyrrolic H), 8.97-8.94 (m, 4H, *β*-pyrrolic H), 8.86 (d, *J* = 4.58 Hz, 2H, *β*-pyrrolic H), 8.79-8.74 (m, 6H, *β*-pyrrolic H), 8.46 (d, *J* = 8.25 Hz, 2H, ArH), 8.26-8.19 (m, 12H, *meso*-ArH), 8.01-7.99 (m, 6H, *meso*-ArH and triazole H), 7.80-7.73 (m, 20H, *meso*-ArH and ArH), 7.69 (d, *J* = 9.17 Hz, 2H, ArH), 7.40 (t, *J* = 7.37 Hz, 4H, *meso*-ArH), 7.25-7.23 (m, 2H, *meso*-ArH), -2.68 (s, 4H, internal NH) ppm; ^{13}C NMR (100 MHz, $CDCl_3$) δ_C: 176.47, 156.71, 145.62, 141.74, 141.62, 141.45, 139.26, 137.11, 134.57, 133.98, 129.71, 129.03, 128.18, 127.95, 127.85, 127.28, 126.82, 126.78, 126.53, 125.32, 121.77, 121.50, 121.22, 120.80, 120.77, 119.34, 114.39 ppm; ESI-MS: m/z = 1577.5 $(M+Na)^+$; Anal. Calcd for $C_{105}H_{66}N_{14}O_2.H_2O$: C, 80.13; H, 4.36; N, 12.46; found: C, 79.92; H, 4.38; N, 12.43.

General procedure for the synthesis of zinc(II) β-triazoloporphyrin–xanthone dyads 6c, 6f and 6i

The zinc(II) porphyrin–xanthone dyads **6c**, **6f** and **6i** were synthesized from the corresponding free-base β-triazoloporphyrin–xanthone dyads by following the literature procedure [8]. The crude products were purified by column chromatography on neutral alumina using 2% methanol in chloroform as eluent.

Zinc(II) 3-amino-6-[1-(5,10,15,20-tetraphenylporphyrin-2-yl)-1*H*-[1,2,3]triazol-4-yl]-xanthen-9-one (6c). Purple solid; yield: 90%; mp >300 °C; UV λ_{max} ($\varepsilon\times10^{-4}$, M^{-1} cm^{-1}): 430 (75.25), 560 (2.70), 600 (1.29) nm; λ_{Em}($CHCl_3$; λ_{Ex} 420 nm): 610, 655 nm;

IR ($CHCl_3$) ν_{max}: 3433, 2918, 2849, 1612, 1435, 1337, 1193, 1070, 1003, 991, 884, 796, 702, 667 cm^{-1}; ^1H NMR (400 MHz, $CDCl_3$ + DMSO-d_6) δ_H: 8.96 (s, 1H, *β*-pyrrolic H), 8.74-8.69 (m, 4H, *β*-pyrrolic H), 8.63 (d, *J* = 4.88 Hz, 1H, *β*-pyrrolic H), 8.49 (d, *J* = 4.88 Hz, 1H, *β*-pyrrolic H), 8.18 (d, *J* = 7.93 Hz, 1H, ArH), 8.09-8.02 (m, 6H, *meso*-ArH), 7.93 (d, *J* = 9.16 Hz, 1H, ArH), 7.79 (d, *J* = 7.93 Hz, 2H, *meso*-ArH), 7.74 (s, 1H, triazole H), 7.71-7.70 (m, 1H, ArH), 7.63-7.55 (m, 9H, *meso*-ArH), 7.48-7.46 (m, 1H, ArH), 7.17 (t, *J* = 7.32 Hz, 2H, *meso*-ArH), 7.03-6.99 (m, 1H, *meso*-ArH), 6.54-6.49 (m, 2H, ArH), 4.89 (brs, 2H, -NH_2) ppm; ^{13}C NMR (100 MHz, $CDCl_3$ + DMSO-d_6) δ_C: 174.90, 158.12, 155.95, 153.70, 150.64, 150.58, 150.54, 150.40, 149.47, 145.18, 143.77, 142.43, 142.29, 140.18, 139.63, 136.50, 135.78, 134.08, 133.33, 132.39, 132.11, 132.06, 131.98, 131.78, 131.49, 131.45, 127.70, 127.19, 127.00, 126.83, 126.45, 126.06, 125.56, 125.29, 121.57, 120.83, 120.74, 120.67, 120.57, 119.20, 113.55, 112.50, 112.21, 98.84 ppm; ESI-MS: m/z = 953.1 $(M+H)^+$; Anal. Calcd for $C_{59}H_{36}N_8O_2Zn.3H_2O$: C, 70.27; H, 4.20; N, 11.11; found: C, 7054; H, 4.38; N, 10.96.

Zinc(II) 3-nitro-6-[1-(5,10,15,20-tetraphenylporphyrin-2-yl)-1*H*-[1,2,3]triazol-4-yl]-xanthen-9-one (6f). Purple solid; yield: 92%; mp >300 °C; UV λ_{max} ($\varepsilon\times10^{-4}$, M^{-1} cm^{-1}): 431 (99.43), 564 (3.51), 604 (1.91) nm; λ_{Em}($CHCl_3$; λ_{Ex} 420 nm): 616, 662 nm; IR (KBr) ν_{max}: 2918, 2849, 1608, 1437, 1341, 1124, 1003, 986, 737, 701 cm^{-1}; ^1H NMR (400 MHz, DMSO-d_6) δ_H: 8.97 (s, 1H, *β*-pyrrolic H), 8.80-8.78 (m, 4H, *β*-pyrrolic H), 8.70 (d, *J* = 3.66 Hz, 1H, *β*-pyrrolic H), 8.53 (d, *J* = 4.27 Hz, 1H, *β*-pyrrolic H), 8.41-8.27 (m, 3H, ArH), 8.21-8.15 (m, 8H, *meso*-ArH and ArH), 8.00 (s, 1H, triazole H), 7.93-7.91 (m, 1H, ArH), 7.87 (d, *J* = 8.54 Hz, *meso*-ArH), 7.81-7.75 (m, 9H, *meso*-ArH), 7.28-7.25 (m, 2H, *meso*-ArH), 7.10-7.06 (m, 1H, *meso*-ArH and ArH) ppm; ^{13}C NMR (100 MHz, DMSO-d_6 + $CDCl_3$) δ_C: 174.34, 150.46, 150.40, 150.30, 150.20, 149.26, 144.24, 143.56, 142.29, 142.17, 139.82, 139.60, 138.24, 137.02, 134.11,

133.49, 132.49, 132.18, 132.12, 131.92, 131.55, 130.96, 127.67, 127.58, 127.41, 126.71, 126.49, 125.53, 122.65, 121.92, 121.46, 120.77, 120.71, 119.94, 119.50, 117.75 ppm; ESI-MS: m/z = 983.3 $(M+H)^+$; Anal. Calcd for $C_{59}H_{34}N_8O_4Zn.H_2O$: C, 70.70; H, 3.62; N, 11.18; found: C, 70.58; H, 3.74; N, 10.94.

Zinc(II) 3-methoxy-6-[1-(5,10,15,20-tetraphenylporphyrin-2-yl)-1*H*-[1,2,3]triazol-4-yl]-xanthen-9-one (6i). Purple solid; yield: 93%; mp >300 °C; UV λ_{max} ($\varepsilon\times10^{-4}$, M^{-1} cm^{-1}): 430 (88.04), 561 (3.10), 602 (1.34) nm; λ_{Em}($CHCl_3$; λ_{Ex} 420 nm): 610, 655 nm; IR ($CHCl_3$) ν_{max}: 3017, 2922, 1602, 1459, 1438, 1338, 1281, 1255, 1194, 1165, 1071, 1034, 1003, 993, 835, 781, 796, 754, 722, 702 cm^{-1}; ^1H NMR (400 MHz, $CDCl_3$) δ_H: 9.02 (s, 1H, *β*-pyrrolic H), 8.95-8.91 (m, 3H, *β*-pyrrolic H), 8.88 (d, *J* = 4.88 Hz, 1H, *β*-pyrrolic H), 8.84 (d, *J* = 4.88 Hz, 1H, *β*-pyrrolic H), 8.66 (d, *J* = 4.88 Hz, 1H, *β*-pyrrolic H), 8.23-8.19 (m, 4H, *meso*-ArH), 8.14 (d, *J* = 6.71 Hz, 2H, *meso*-ArH), 7.83 (d, *J* = 7.32 Hz, 2H, *meso*-ArH), 7.78-7.66 (m, 10H, *meso*-ArH and ArH), 7.57 (s, 1H, triazole H), 7.32-7.27 (m, 2H, ArH), 7.24-7.20 (m, 2H, *meso*-ArH), 7.17-7.15 (m, 1H, ArH), 7.08-7.05 (m, 1H, *meso*-ArH), 6.78-6.73 (m, 2H, ArH), 3.90 (s, 3H, $-OCH_3$) ppm; ^{13}C NMR (100 MHz, $CDCl_3$ + DMSO-d_6) δ_C: 174.69, 164.21, 157.25, 155.61, 150.08, 150.01, 149.87, 148.92, 144.39, 143.21, 141.84, 141.74, 139.63, 139.11, 136.03, 133.54, 132.83, 131.91, 131.62, 131.53, 131.31, 130.97, 130.87, 127.02, 126.76, 126.58, 126.29, 126.00, 125.63, 125.27, 125.04, 121.05, 120.53, 120.26, 120.18, 120.03, 118.70, 114.80, 113.15, 112.53, 99.45, 55.07 ppm; ESI-MS: m/z = 1006.2 $(M+K)^+$; Anal. Calcd for $C_{60}H_{37}N_7O_3Zn$: C, 72.98; H, 3.98; N, 9.93; found: C, 73.13; H, 3.89; N, 9.86.

General procedure for the synthesis of zinc(II) β-triazolomethylporphyrin–xanthone dyads (7a, 7c) and zinc(II) xanthone-bridged β-triazolomethyldiporphyrin 13a

To a solution of zinc(II) 2-azidomethyl-5,10,15,20-tetraphenylporphyrin (100 mg, 0.136 mmol) in DMF (10 mL), $CuSO_4 \cdot 5H_2O$ (6.79 mg, 0.027 mmol), ascorbic acid (9.58 mg, 0.054 mmol) and ethynyl-substituted xanthones (**3–5** 0.163 mmol or **11**, 0.081 mmol) were added. The reaction mixture was stirred at 80 °C for 2–4 h. After completion of the reaction as indicated by TLC, the mixture was allowed to cool to rt and ~50 mL chloroform were added. The resulting solution was washed thoroughly with water, the organic layer was dried over anhydrous sodium sulfate and the solvent was evaporated under reduced pressure. The crude product was purified by column chromatography over activated neutral alumina using 1% methanol in chloroform as the eluent.

Zinc(II) 3-amino-6-[1-(5,10,15,20-tetraphenylporphyrin-2-ylmethyl)-1*H*-[1,2,3]-triazol-4-yl]-xanthen-9-one (7a). Purple solid; yield: 69%; mp >300 °C; UV λ_{max} ($\varepsilon \times 10^{-4}$, M^{-1} cm^{-1}): 427 (83.48), 556 (3.22), 600 (0.77) nm; λ_{Em}($CHCl_3$; λ_{Ex} 420 nm): 607, 652 nm; IR ($CHCl_3$) ν_{max}: 3384, 2918, 1602, 1432, 1271, 1254, 1190, 1123, 1067, 1002, 991, 853, 792, 751, 701 cm^{-1}; ^{1}H NMR (400 MHz, DMSO-d_6) δ_H: 8.83 (s, 1H, *β*-pyrrolic H), 8.77-8.72 (m, 5H, *β*-pyrrolic H), 8.61 (d, *J* = 4.27 Hz, 1H, *β*-pyrrolic H), 8.18-8.15 (m, 8H, *meso*-ArH), 8.08-8.06 (m, 2H, ArH), 7.94 (s, 1H, triazole H), 7.86 (d, *J* = 7.93 Hz, 2H, ArH), 7.82-7.79 (m, 9H, *meso*-ArH), 7.62-7.60 (m, 3H, *meso*-ArH), 6.68-6.66 (m, 1H, ArH), 6.54-6.52 (m, 3H, ArH and -NH_2), 5.76 (s, 2H, -CH_2) ppm; ^{13}C NMR (100 MHz, DMSO-d_6) δ_C: 173.54, 158.20, 155.88, 150.12, 149.71, 149.58, 149.53, 149.42, 146.84, 145.20, 144.94, 142.63, 142.51, 142.40, 139.47, 136.18, 134.28, 134.24, 132.93, 132.63, 132.15, 131.98, 131.86, 131.42,

128.64, 127.63, 127.41, 126.73, 126.44, 123.84, 121.01, 120.86, 120.75, 120.60, 120.02, 119.88, 113.50, 112.59, 110.91, 97.59 ppm; ESI-MS: m/z = 967.3 $(M+H)^+$; Anal. Calcd for $C_{60}H_{38}N_8O_2Zn.2H_2O$: C, 71.75; H, 4.21; N, 11.16; found: C, 71.74; H, 4.22; N, 11.21.

Zinc(II) 3-methoxy-6-[1-(5,10,15,20-tetraphenylporphyrin-2-ylmethyl)-1*H*-[1,2,3]-triazol-4-yl]-xanthen-9-one (7c). Purple solid; yield: 76%; mp >300 °C; UV λ_{max} ($\varepsilon \times 10^{-4}$, M^{-1} cm^{-1}): 427 (85.02), 557 (3.95), 599 (0.99) nm; λ_{Em}($CHCl_3$; λ_{Ex} 420 nm): 607, 651 nm; IR ($CHCl_3$) ν_{max}: 2925, 1606, 1437, 1282, 1259, 1193, 1121, 1066, 1004, 994, 851, 794, 752, 700 cm^{-1}; ^1H NMR (400 MHz, DMSO-d_6) δ_H: 8.83 (s, 1H, *β*-pyrrolic H), 8.76-8.71 (m, 5H, *β*-pyrrolic H), 8.60 (d, *J* = 4.88 Hz, 1H, *β*-pyrrolic H), 8.21 (d, *J* = 7.93 Hz, 1H, ArH), 8.17-8.13 (m, 6H, *meso*-ArH), 8.09 (d, *J* = 9.16 Hz, 1H, ArH), 8.06-8.03 (m, 2H, *meso*-ArH), 7.99 (s, 1H, triazole H), 7.90-7.84 (m, 2H, ArH), 7.81-7.78 (m, 9H, *meso*-ArH), 7.59-7.58 (m, 3H, *meso*-ArH), 7.11 (s, 1H, ArH), 7.04 (dd, J_1 = 8.54 and J_2 = 1.83 Hz, 1H, ArH), 5.76 (s, 2H, -CH_2), 3.90 (s, 3H, -OCH_3) ppm; ^{13}C NMR (100 MHz, DMSO-d_6 + $CDCl_3$) δ_C: 174.50, 164.89, 157.65, 156.08, 150.06, 149.65, 149.51, 149.48, 149.37, 149.32, 146.74, 144.91, 144.81, 142.55, 142.44, 142.35, 139.21, 136.94, 134.17, 134.11, 132.84, 132.58, 131.97, 131.80, 131.67, 131.23, 128.44, 127.53, 127.40, 127.23, 126.73, 126.50, 126.23, 123.80, 121.18, 120.83, 120.42, 119.82, 119.76, 115.10, 113.49, 100.47, 56.04 ppm; ESI-MS: m/z = 1004.2 $(M+Na)^+$; Anal. Calcd for $C_{61}H_{39}N_7O_3Zn.H_2O$: C, 73.16; H, 4.13; N, 9.79; found: C, 73.24; H, 3.98; N, 9.83.

Zinc(II) 3,6-bis-[1-(5,10,15,20-tetraphenylporphyrin-2-ylmethyl)-1*H*-[1,2,3]triazol-4-yl]-xanthen-9-one (13a). Purple solid; yield: 39%; mp >300 °C; UV λ_{max} ($\varepsilon \times 10^{-4}$, M^{-1} cm^{-1}): 426 (180.92), 557 (7.63), 578 (2.45) nm; λ_{Em}($CHCl_3$; λ_{Ex} 420 nm): 608, 654

nm; IR ($CHCl_3$) ν_{max}: 3055, 3019, 1654, 1615, 1597, 1483, 1439, 1338, 1217, 1122, 1070, 1004, 994, 796, 753, 701 cm^{-1}; ^{1}H NMR (400 MHz, DMSO-d_6): δ_H: 8.88-8.70 (m, 12H, *β*-pyrrolic H), 8.60 (d, *J* = 4.27 Hz, 2H, *β*-pyrrolic H), 8.37-8.26 (m, 2H, ArH), 8.19-8.12 (m, 14H, *meso*-ArH and ArH), 8.08-8.03 (m, 4H, *meso*-ArH), 7.94 (s, 2H, triazole H), 7.87-7.83 (m, 2H, ArH), 7.81-7.75 (m, 18H, *meso*-ArH), 7.62-7.56 (m, 6H, *meso*-ArH), 5.76 (s, 4H, -CH_2); ^{13}C NMR (100 MHz, DMSO-d_6 + $CDCl_3$) δ_C: 171.92, 150.04, 149.62, 149.50, 149.45, 149.34, 149.30, 146.72, 144.80, 142.54, 142.41, 142.33, 139.17, 137.33, 134.15, 134.10, 132.81, 132.58, 131.96, 131.79, 131.67, 131.23, 128.42, 127.39, 127.21, 126.49, 126.22, 123.96, 120.82, 120.44, 119.80, 119.75, 49.57 ppm; ESI-MS: m/z = 1707.4 $(M+H)^+$; Anal. Calcd for $C_{107}H_{66}N_{14}O_2Zn_2.H_2O$: C, 74.35; H, 3.97; N, 11.34; found: C, 74.29; H, 3.98; N, 11.36.

General procedure for the synthesis of free-base β-triazolomethylporphyrin–xanthone dyads (7b, 7d) and xanthone-bridged β-triazolomethyldiporphyrin 13b

The free-base porphyrins **7b**, **7d** and **13b** were synthesized from the corresponding zinc(II) porphyrin–xanthone dyads by using the reported procedure [9]. The crude products were purified over neutral alumina using chloroform as eluent.

3-Amino-6-[1-(5,10,15,20-tetraphenylporphyrin-2-ylmethyl)-1*H*-[1,2,3]triazol-4-yl]-xanthen-9-one (7b). Purple solid; yield: 82%; mp >300 °C; UV λ_{max} ($\varepsilon\times10^{-4}$, M^{-1} cm^{-1}): 421 (60.82), 519 (2.30), 553 (0.88), 594 (0.34), 649 (0.30) nm; λ_{Em}($CHCl_3$; λ_{Ex} 420 nm): 657, 720 nm; IR ($CHCl_3$) ν_{max}: 3431, 3327, 3216, 2922, 1615, 1598, 1457, 1440, 1357, 1339, 1311, 1189, 1120, 966, 797, 751, 699 cm^{-1}; ^{1}H NMR (400 MHz, DMSO-d_6) δ_H: 8.84-8.80 (m, 5H, *β*-pyrrolic H), 8.74 (s, 1H, *β*-pyrrolic H), 8.67 (d, *J* =

4.58 Hz, 1H, *β*-pyrrolic H), 8.28 (s, 1H, triazole H), 8.22-8.20 (m, 4H, *meso*-ArH), 8.16-8.10 (m, 5H, *meso*-ArH and ArH), 7.88-7.77 (m, 12H, ArH and *meso*-ArH), 7.68-7.65 (m, 3H, *meso*-ArH), 6.64 (dd, J_1 = 9.17 and J_2 = 1.83 Hz, 1H, ArH), 6.51 (s, 2H, -NH_2), 6.47 (d, *J* = 1.83 Hz, 1H, ArH), 5.78 (s, 2H, -CH_2), -2.87 (s, 2H, internal NH); ^{13}C NMR (100 MHz, DMSO-d_6) δ_C: 173.40, 158.08, 155.77, 145.06, 141.18, 140.87, 140.47, 137.30, 136.01, 134.27, 134.20, 132.94, 129.10, 128.19, 128.05, 127.64, 127.54, 127.13, 127.03, 126.77, 126.54, 123.68, 120.77, 120.62, 120.56, 120.24, 119.64, 113.40, 112.49, 110.79, 97.46 ppm; ESI-MS: m/z = 905.2 $(M+H)^+$; Anal. Calcd for $C_{60}H_{40}N_8O_2.H_2O$: C, 78.04; H, 4.59; N, 12.14; found: C, 78.16; H, 4.36; N, 11.94.

3-Methoxy-6-[1-(5,10,15,20-tetraphenylporphyrin-2-ylmethyl)-1*H*-[1,2,3]triazol-4-yl]-xanthen-9-one (7d). Purple solid; yield: 83%; mp >300 °C; UV λ_{max} ($\varepsilon\times10^{-4}$, M^{-1} cm^{-1}): 421 (63.37), 519 (2.41), 552 (1.05), 593 (0.41), 649 (0.38) nm; λ_{Em}($CHCl_3$; λ_{Ex} 420 nm): 657, 720 nm; IR ($CHCl_3$) ν_{max}: 3324, 3055, 2921, 2848, 1612, 1437, 1351, 1268, 1253, 1199, 1102, 1001, 964, 799, 752, 702 cm^{-1}; ^{1}H NMR (400 MHz, $CDCl_3$) δ_H: 8.89-8.80 (m, 5H, *β*-pyrrolic H), 8.67 (d, *J* = 4.88 Hz, 1H, *β*-pyrrolic H), 8.48 (s, 1H, *β*-pyrrolic H), 8.22-8.16 (m, 6H, *meso*-ArH), 8.15-8.13 (m, 2H, ArH), 8.05 (d, *J* = 6.71 Hz, 2H, *meso*-ArH), 7.85-7.70 (m, 10H, ArH and *meso*-ArH), 7.68-7.65 (m, 3H, *meso*-ArH), 7.52 (s, 1H, triazole H), 7.47 (dd, J_1 = 8.54 and J_2 = 1.22 Hz, 1H, ArH), 6.89 (dd, J_1 = 9.16 and J_2 = 2.44 Hz, 1H, ArH), 6.79 (d, *J* = 2.44 Hz, 1H, ArH), 5.82 (s, 2H, CH_2), 3.88 (s, 3H, OCH_3), -2.72 (s, 2H, internal NH) ppm; ^{13}C NMR (100 MHz, $CDCl_3$) δ_C: 175.72, 164.95, 158.01, 156.44, 145.65, 141.89, 141.77, 141.61, 141.11, 136.48, 134.57, 134.54, 134.51, 133.12, 130.64, 130.18, 128.94, 128.08, 127.97, 127.84, 127.59, 127.07, 126.81, 126.72, 126.65, 121.35, 121.09, 121.04, 120.74, 120.56, 120.21, 119.26, 115.71, 114.01, 113.21, 100.07, 55.77, 49.88 ppm;

ESI-MS: m/z = 920.3 $(M+H)^+$; Anal. Calcd for $C_{61}H_{39}CuN_7O_3$: C, 74.64; H, 4.00; N, 9.99; found: C, 74.71; H, 3.96; N, 9.92.

3,6-Bis[1-(5,10,15,20-tetraphenylporphyrin-2-ylmethyl)-1*H*-[1,2,3]triazol-4-yl]-xanthen-9-one (**13b**). Purple solid; yield: 84%; mp >300 °C; UV λ_{max} ($\varepsilon \times 10^{-4}$, M^{-1} cm^{-1}): 421 (162.20), 518 (6.78), 552 (2.51), 594 (1.72), 649 (1.32) nm; λ_{Em}($CHCl_3$; λ_{Ex} 420 nm): 656, 719 nm; IR ($CHCl_3$) ν_{max}: 3326, 3056, 2921, 2850, 1618, 1438, 1350, 1220, 1178, 1044, 1001, 965, 799, 753, 702 cm^{-1}; ^1H NMR (400 MHz, $CDCl_3$) δ_H: 8.87-8.81 (m, 10H, *β*-pyrrolic H), 8.66 (d, *J* = 4.58 Hz, 2H, *β*-pyrrolic H), 8.44 (s, 2H, *β*-pyrrolic H), 8.21-8.11 (m, 14H, *meso*-ArH and ArH), 8.04 (d, *J* = 7.34 Hz, 4H, *meso*-ArH), 7.84-7.69 (m, 20H, ArH and *meso*-ArH), 7.64-7.62 (m, 6H, *meso*-ArH), 7.52-7.48 (m, 4H, triazole H and ArH), 5.80 (s, 4H, $-CH_2$), -2.73 (s, 4H, internal NH) ppm; ^{13}C NMR (100 MHz, $CDCl_3$) δ_C: 176.09, 156.43, 145.54, 141.89, 141.75, 141.60, 141.11, 136.92, 134.56, 134.50, 133.10, 132.38, 130.46, 130.25, 128.93, 127.92, 127.83, 127.58, 127.07, 126.79, 126.71, 126.63, 121.43, 121.17, 120.98, 120.72, 120.55, 120.17, 119.25, 114.18, 49.89 ppm; m/z = 1583.5 $(M+H)^+$; Anal. Calcd for $C_{107}H_{70}N_{14}O_2.H_2O$: C, 80.23; H, 4.53; N, 12.24; found: C, 79.98; H, 4.50; N, 12.16.

General procedure for the synthesis of copper(II) β-triazolomethylporphyrin–xanthone dyad (7e) and copper(II) xanthone-bridged β-triazolomethyldiporphyrin 13c

The copper(II) porphyrins **7e** and **13c** were prepared in a similar manner as described earlier [8] by the reaction of free-base porphyrin **7d** (20 mg, 0.02 mmol) or free-base diporphyrin **13b** (20 mg, 0.012 mmol) in a mixture of chloroform (10 mL) and methanol (1.0 mL) in the presence of $Cu(OAc)_2 \cdot 2H_2O$ (0.037 mmol). The crude

product was purified over neutral alumina by using 1% methanol in $CHCl_3$ as solvent.

Copper(II) 3-methoxy-6-[1-(5,10,15,20-tetraphenylporphyrin-2-ylmethyl)-1*H*-[1,2,3]-triazol-4-yl]-xanthen-9-one (7e). Purple solid; yield: 91%; mp 260-262 °C; UV λ_{max} ($\varepsilon \times 10^{-4}$, M^{-1} cm^{-1}): 418 (97.86), 542 (4.74), 573 (1.07) nm; IR ($CHCl_3$) ν_{max}: 3054, 3019, 2849, 1653, 1613, 1457, 1438, 1344, 1268, 1253, 1176, 1072, 1005, 996, 798, 754, 702 cm^{-1}; ESI-MS: m/z = 1019.2 $(M+K)^+$; Anal. Calcd for $C_{61}H_{39}CuN_7O_3$: C, 74.64; H, 4.00; N, 9.99; found: C, 74.71; H, 3.96; N, 9.92.

Copper(II) 3,6-bis-[1-(5,10,15,20-tetraphenylporphyrin-2-ylmethyl)-1*H*-[1,2,3]-triazol-4-yl]-xanthen-9-one (**13c**).

Purple solid; yield: 89%; mp >300 °C; UV λ_{max} ($\varepsilon \times 10^{-4}$, M^{-1} cm^{-1}): 418 (168.44), 542 (7.33), 573 (1.03) nm; IR ($CHCl_3$) ν_{max}: 3413, 3057, 2925, 1618, 1438, 1344, 1179, 1072, 1005, 798, 754, 702 cm^{-1}; ESI-MS: m/z = 1705.4 $(M+H)^+$; Anal. Calcd for $C_{107}H_{66}Cu_2N_{14}O_2$: C, 75.29; H, 3.90; N, 11.49; found: C, 75.19; H, 4.10; N, 11.53.

References

1. Li, J.; Hu, M.; Yao, S. Q. *Org. Lett.* **2009**, *11*, 3008-3011.
2. Li, J.; Yao, S. Q. *Org. Lett.* **2009**, *11*, 405-408.
3. Chang, Y-T.; Ahn, Y-H. (29 May 2008), U.S. Pat. Appl. Publ., 20080124751.
4. Šebej, P.; Wintner, J.; Müller, P.; Slanina, T.; Anshori, J. Al; Antony, L. A. P.; Klán, P.; Wirz, J. *J. Org. Chem*. **2013**, *78*, 1833-1843.
5. Piazzi, L.; Belluti, F.; Bisi, A.; Gobbi, S.; Rizzo, S.; Bartolini, M.; Andrisano, V.; Recanatini, M.; Rampa, A. *Bioorg. Med. Chem.* **2007**, *15*, 575-585.
6. Wu, L.; Burgess, K. *Org. Lett*. **2008**, *10*, 1779-1782.
7. Promarak, V.; Burn, P. L. *J. Chem. Soc., Perkin Trans. 1*, **2001**, 14-20
8. Sharma, S.; Nath, M. *New J. Chem.* **2011**, *35*, 1630-1639.

9. Singh, D. K.; Nath, M. *Dyes Pigm.* **2015**, *121*, 256-264.
