# Supplementary material for: Synthesis and spectroscopic properties of β-triazoloporphyrin–xanthone dyads
Source: Beilstein J Org Chem. 2015 Aug 17;11:1434–40. doi: 10.3762/bjoc.11.155 (PMC4578393; doi:10.3762/bjoc.11.155)
Supplement: File 2 — 1H and 13C NMR spectra of newly synthesized compounds. [file Beilstein_J_Org_Chem-11-1434-s002.pdf]

Supporting information File 2

for

Synthesis and spectroscopic properties of β-triazoloporphyrin–xanthone dyads

Dileep Kumar Singh and Mahendra Nath*

Address: Department of Chemistry, University of Delhi, Delhi 110 007, India

Email: Mahendra Nath* - mnath@chemistry.du.ac.in

*Corresponding author

^{1}H and ^{13}C NMR spectra of newly synthesized compounds

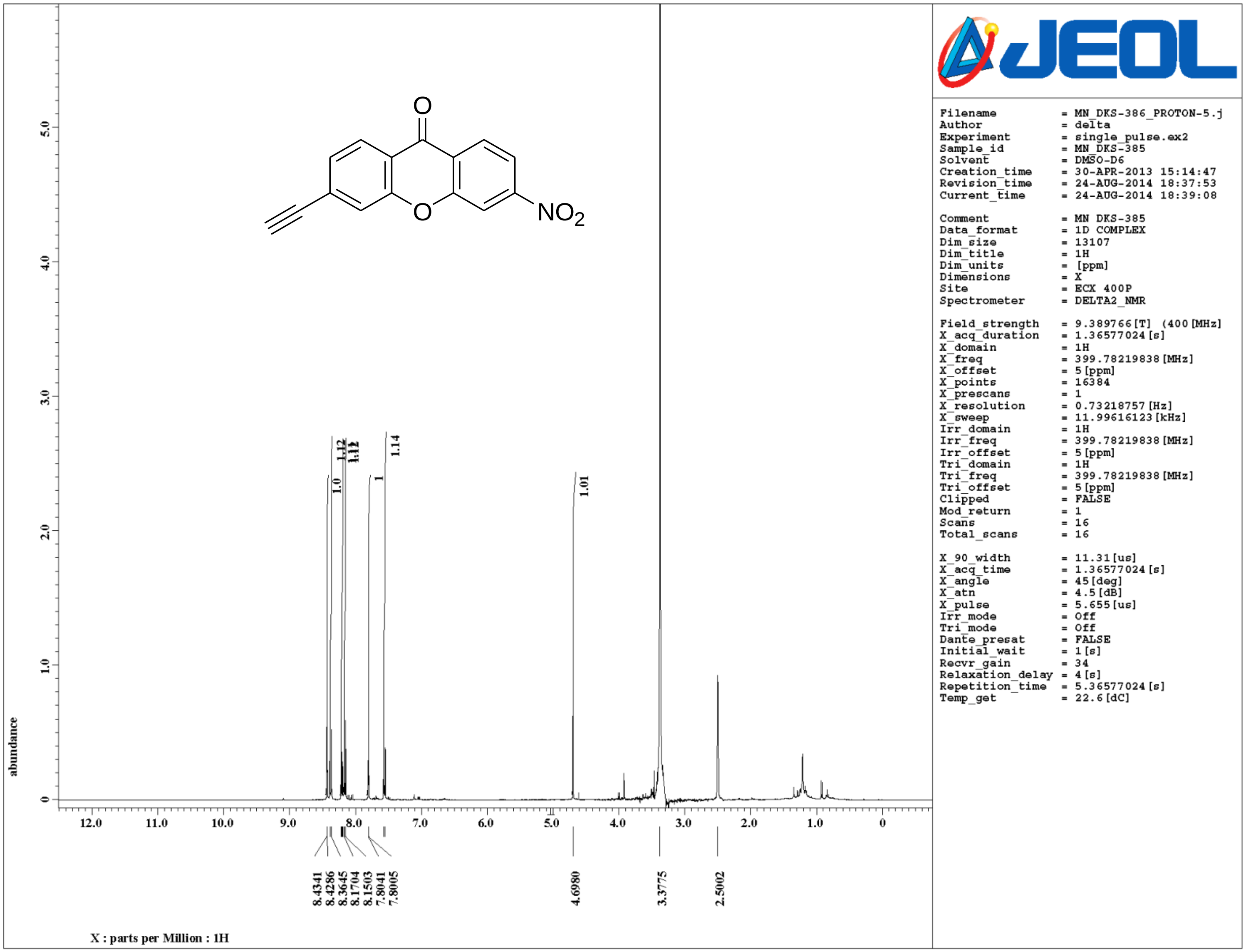

Figure S1: ^{1}H NMR spectrum of compound **4** in DMSO-d_6.

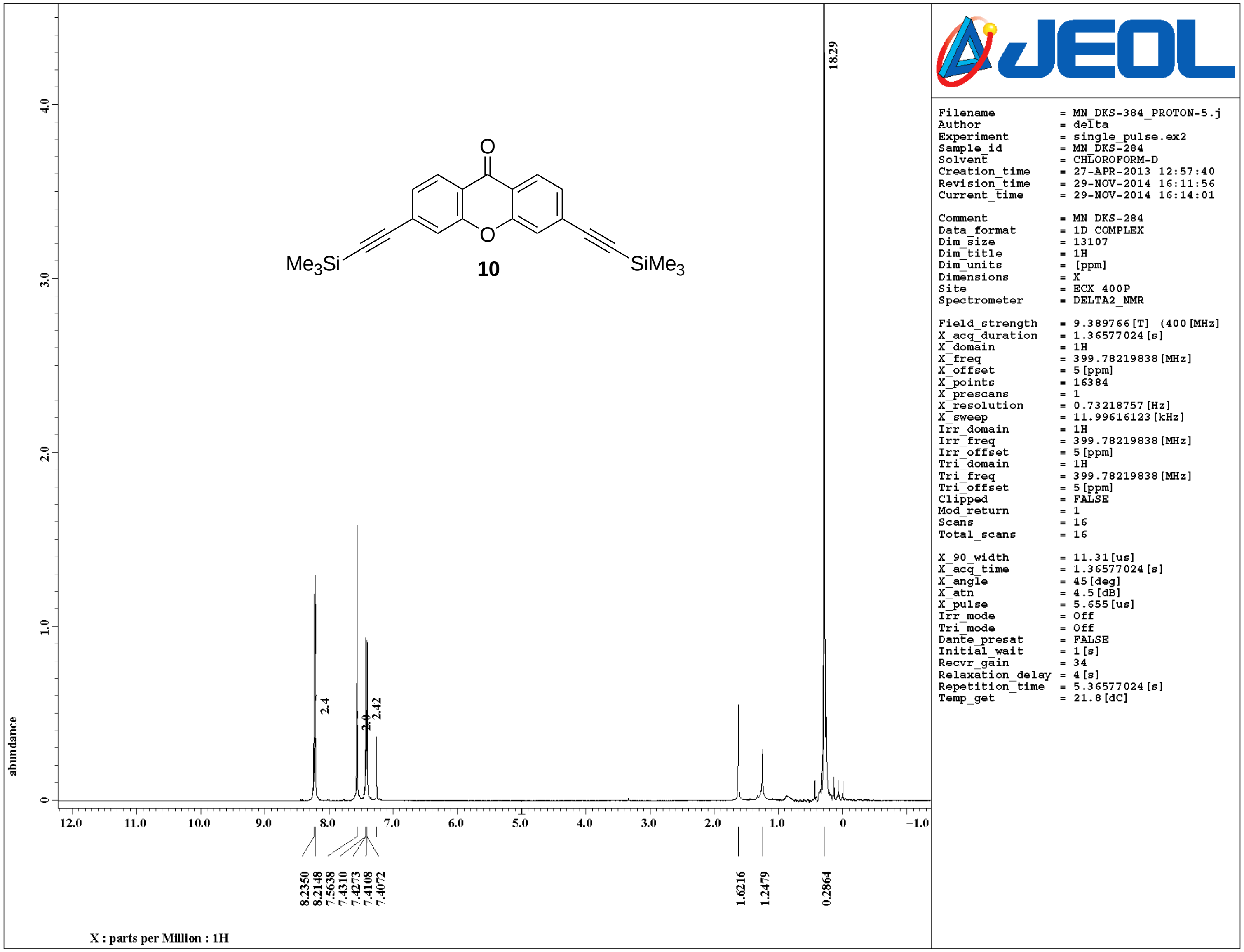

Figure S2: ^{1}H NMR spectrum of compound **10** in $CDCl_3$.

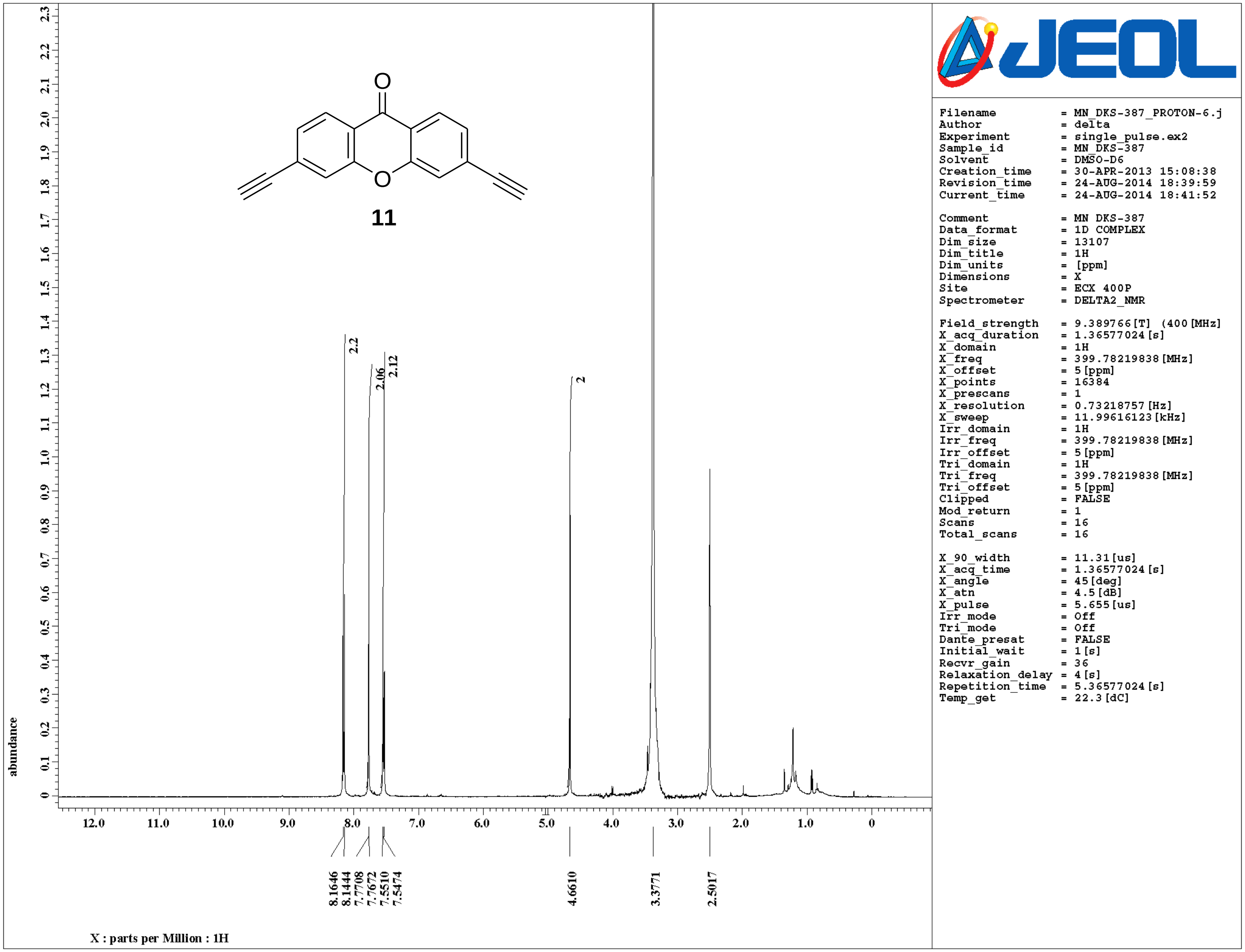

Figure S3: ^{1}H NMR spectrum of compound **11** in DMSO-d_6.

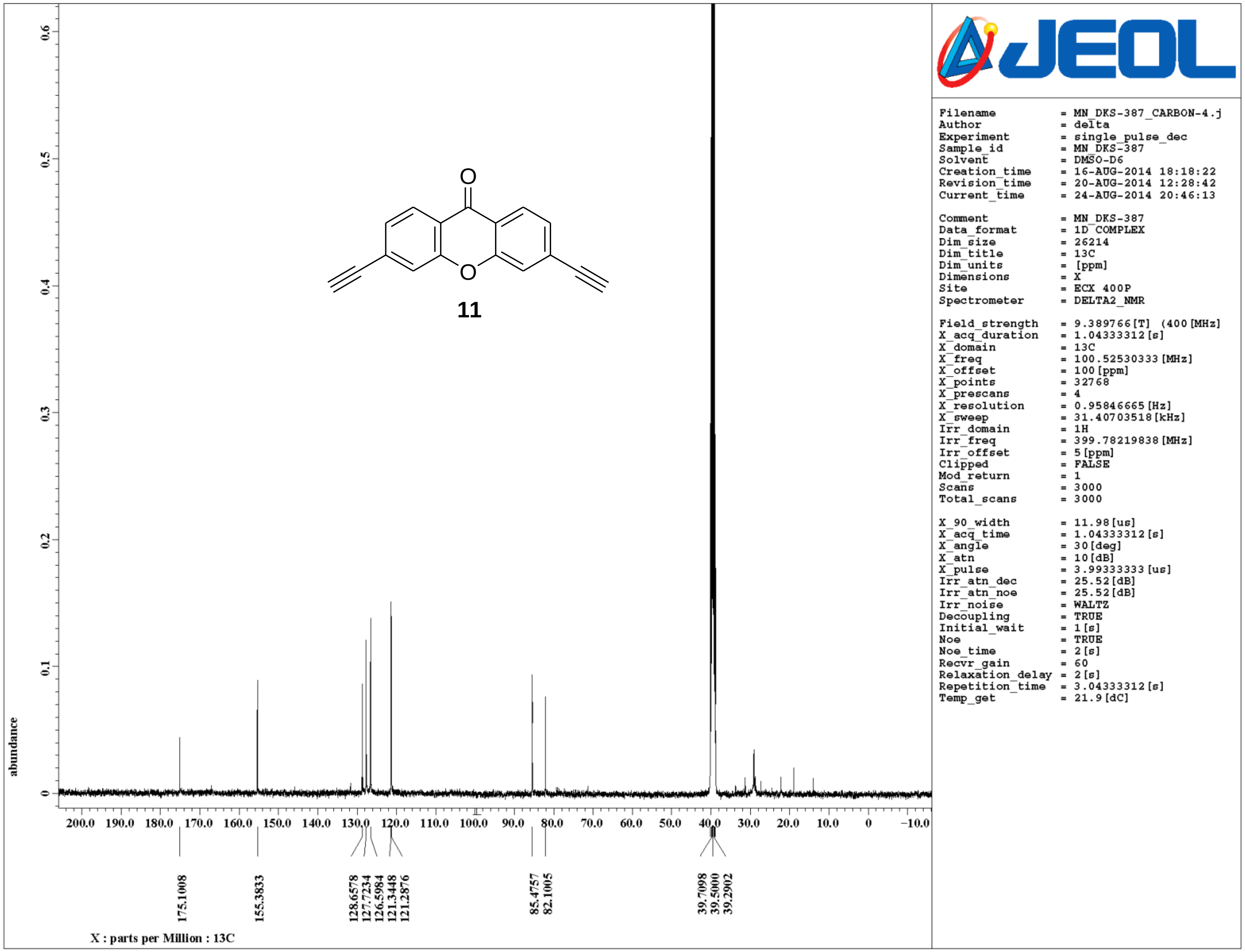

Figure S4: ^{13}C NMR spectrum of compound **11** in DMSO-d_6.

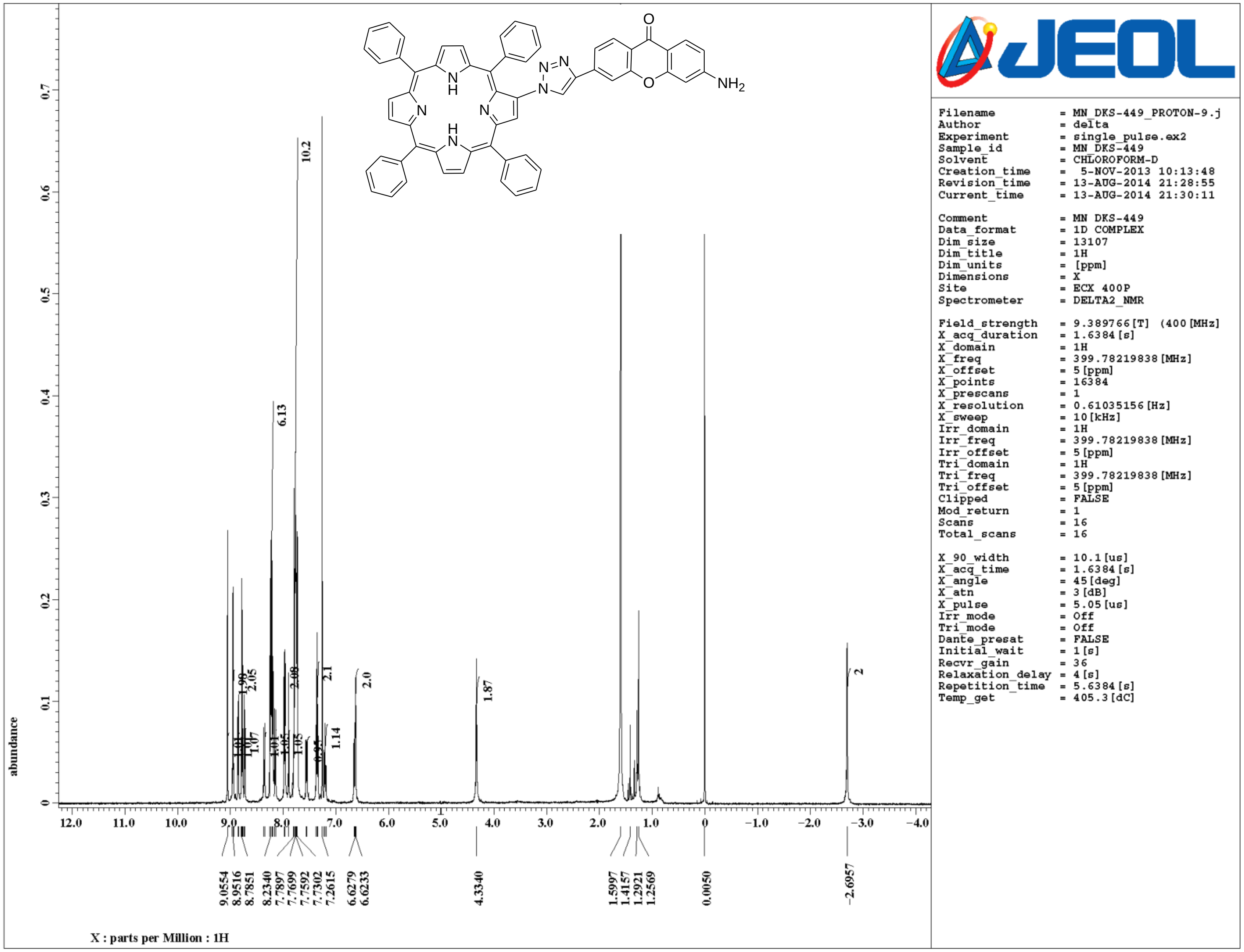

Figure S5: ^{1}H NMR spectrum of compound **6b** in $CDCl_3$.

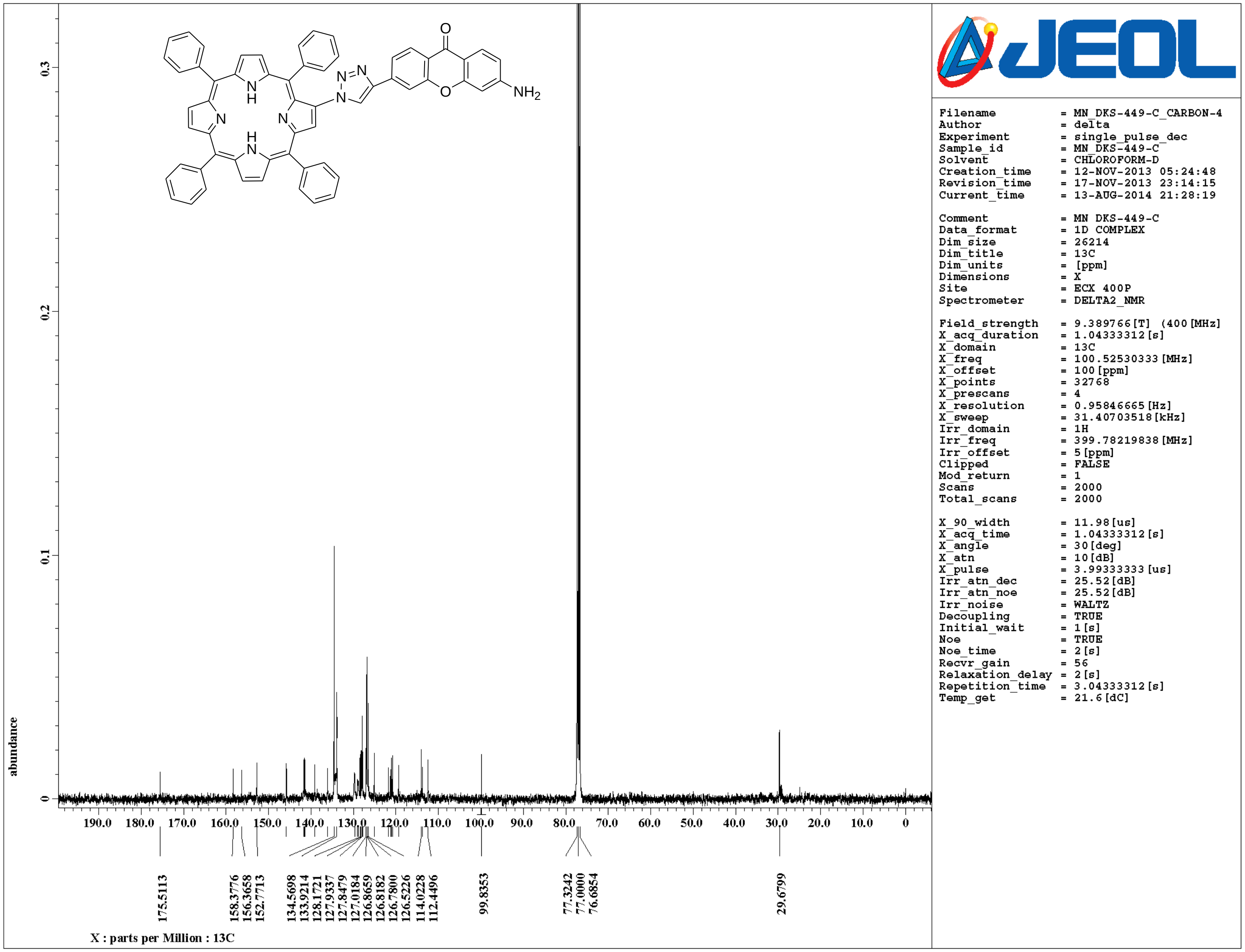

Figure S6: ^{13}C NMR spectrum of compound **6b** in $CDCl_3$.

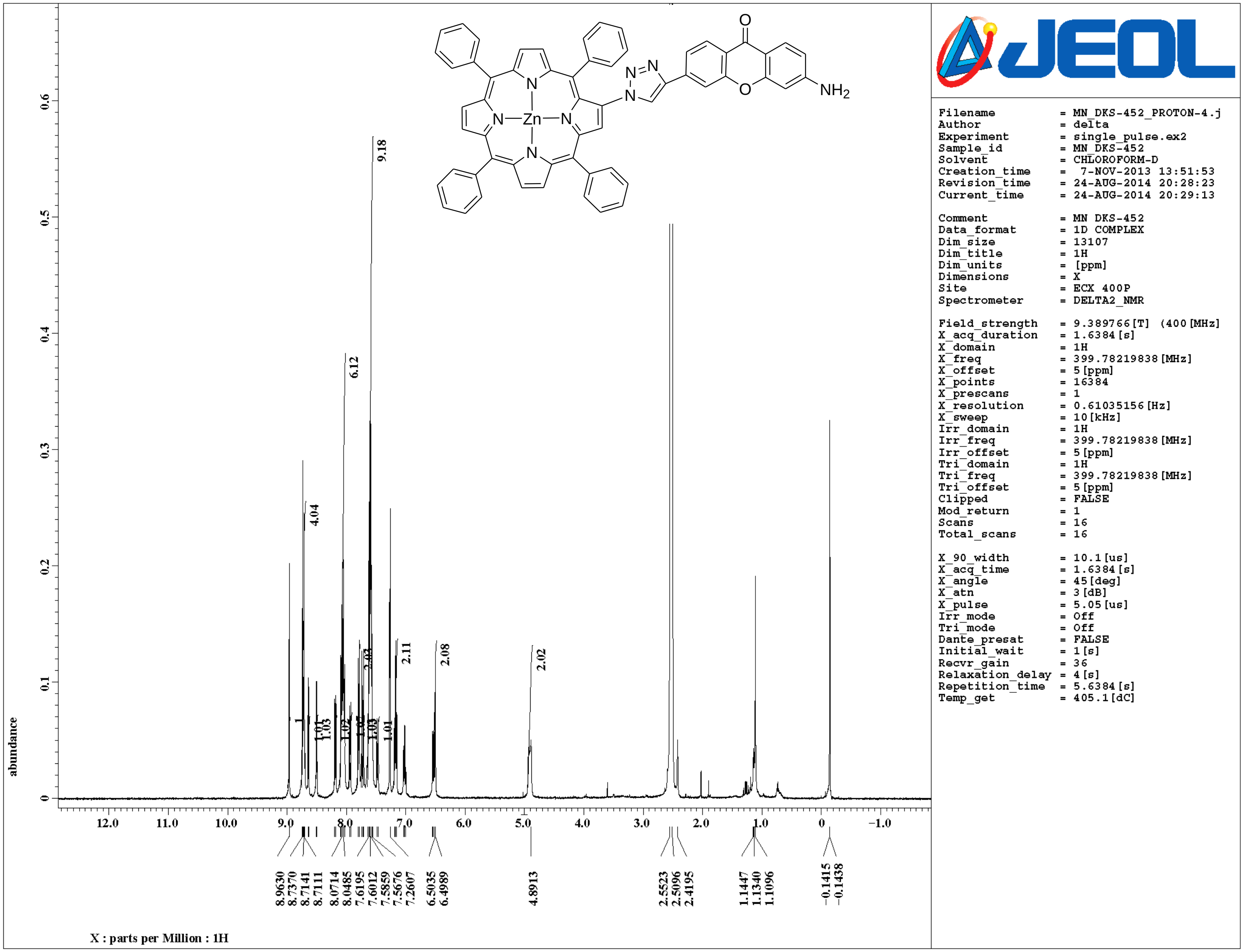

Figure S7: ^{1}H NMR spectrum of compound **6c** in $CDCl_3$ + DMSO-d_6.

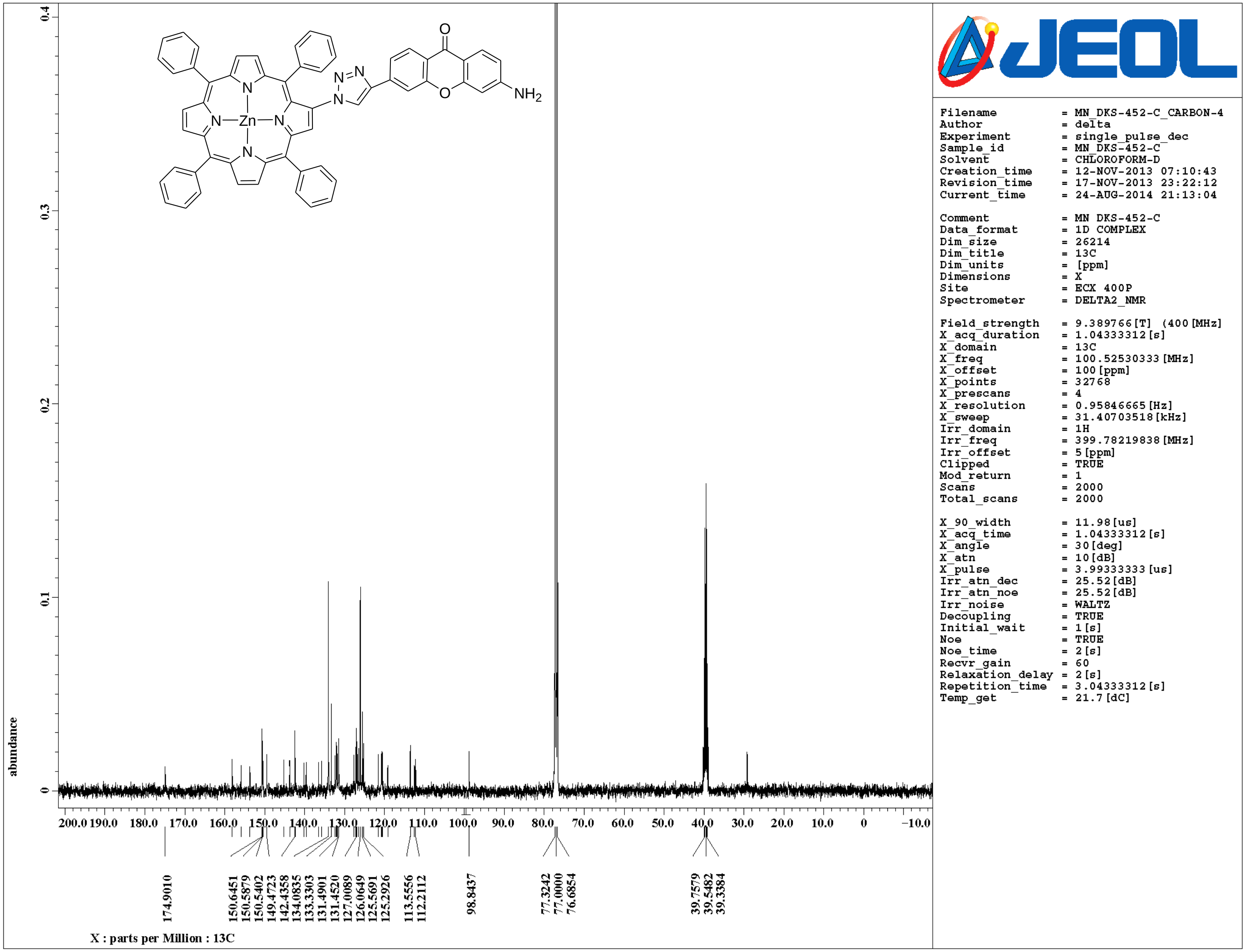

Figure S8: ^{13}C NMR spectrum of compound **6c** in $CDCl_3$ + DMSO-d_6.

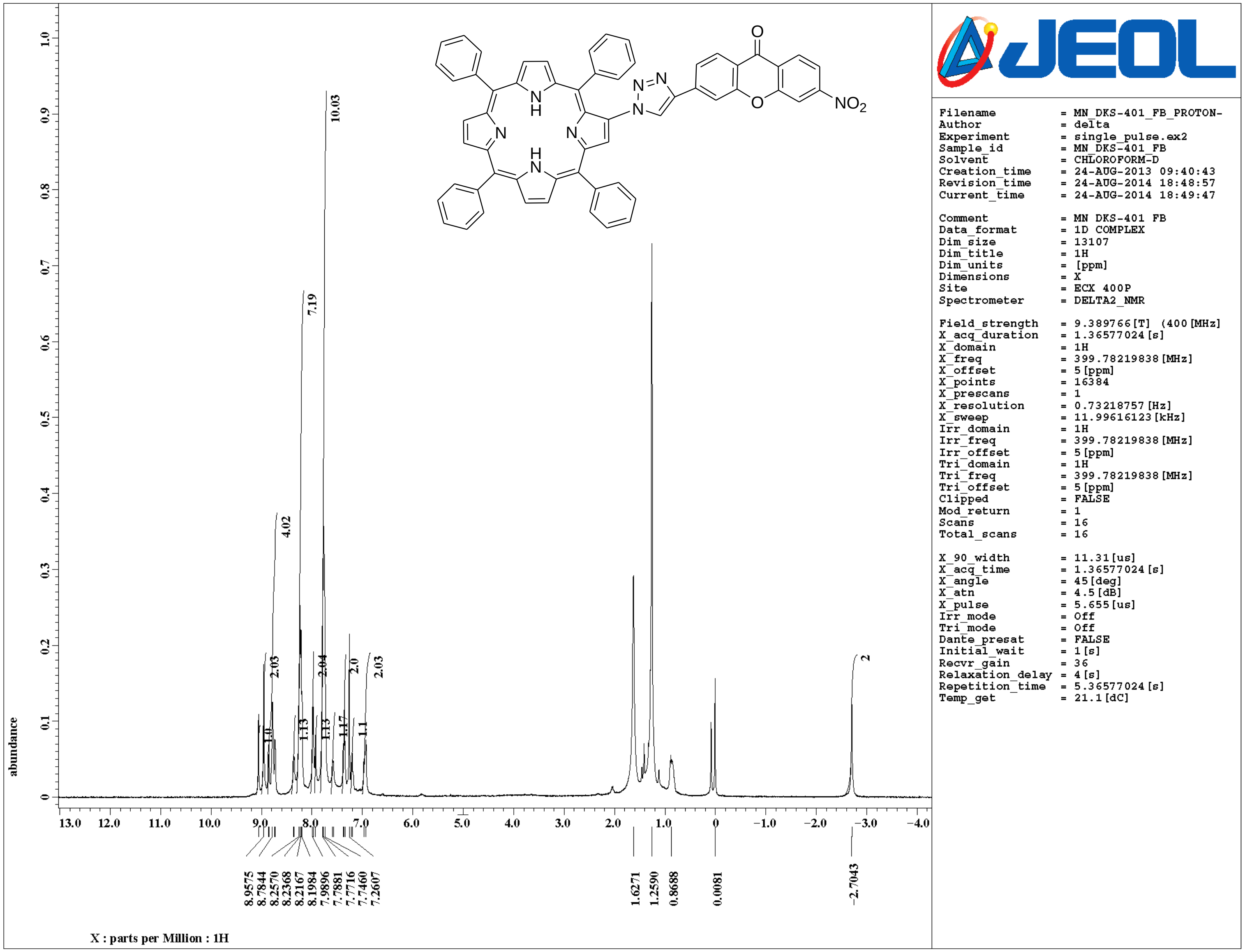

Figure S9: ^{1}H NMR spectrum of compound **6e** in $CDCl_3$.

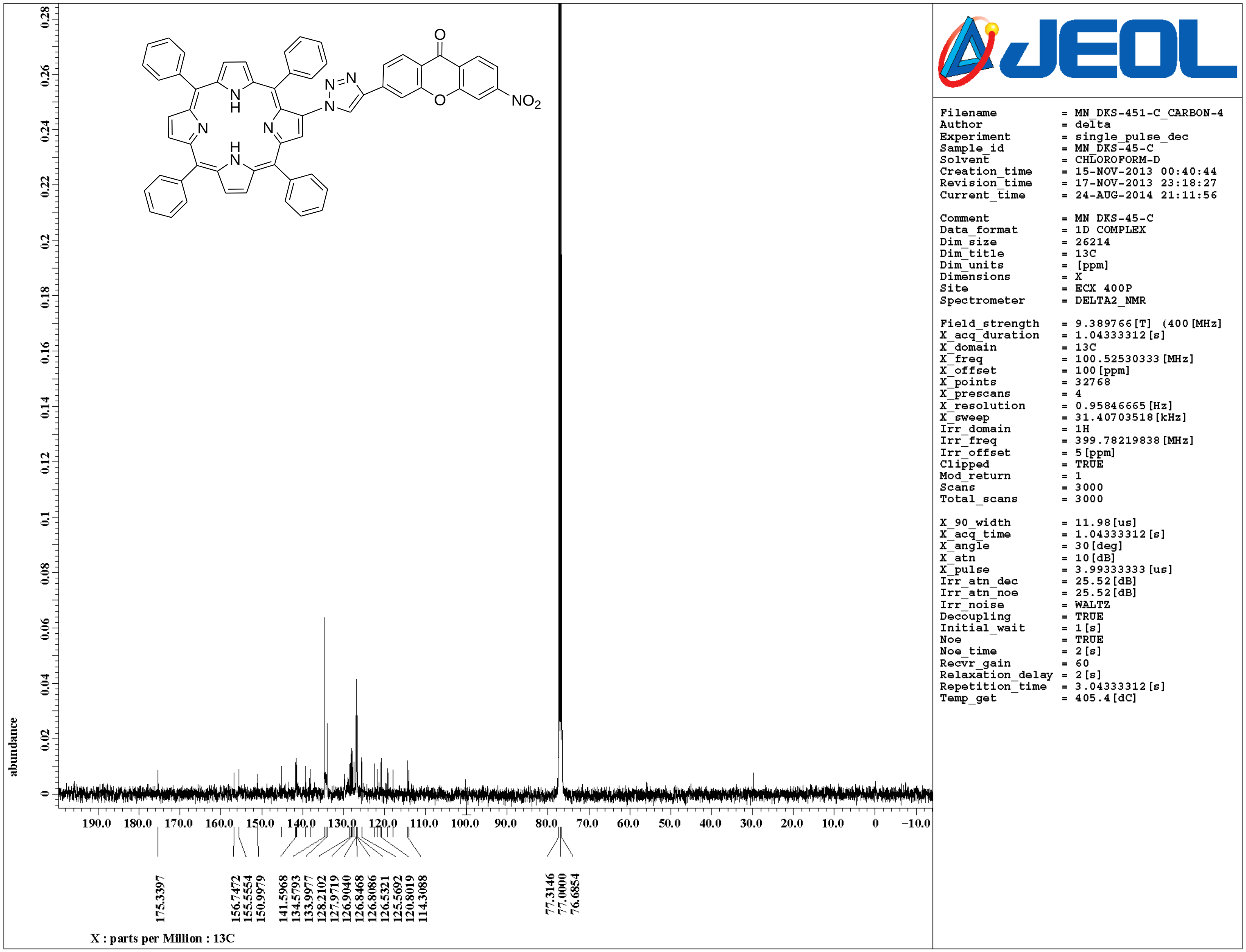

Figure S10: ^{13}C NMR spectrum of compound **6e** in $CDCl_3$.

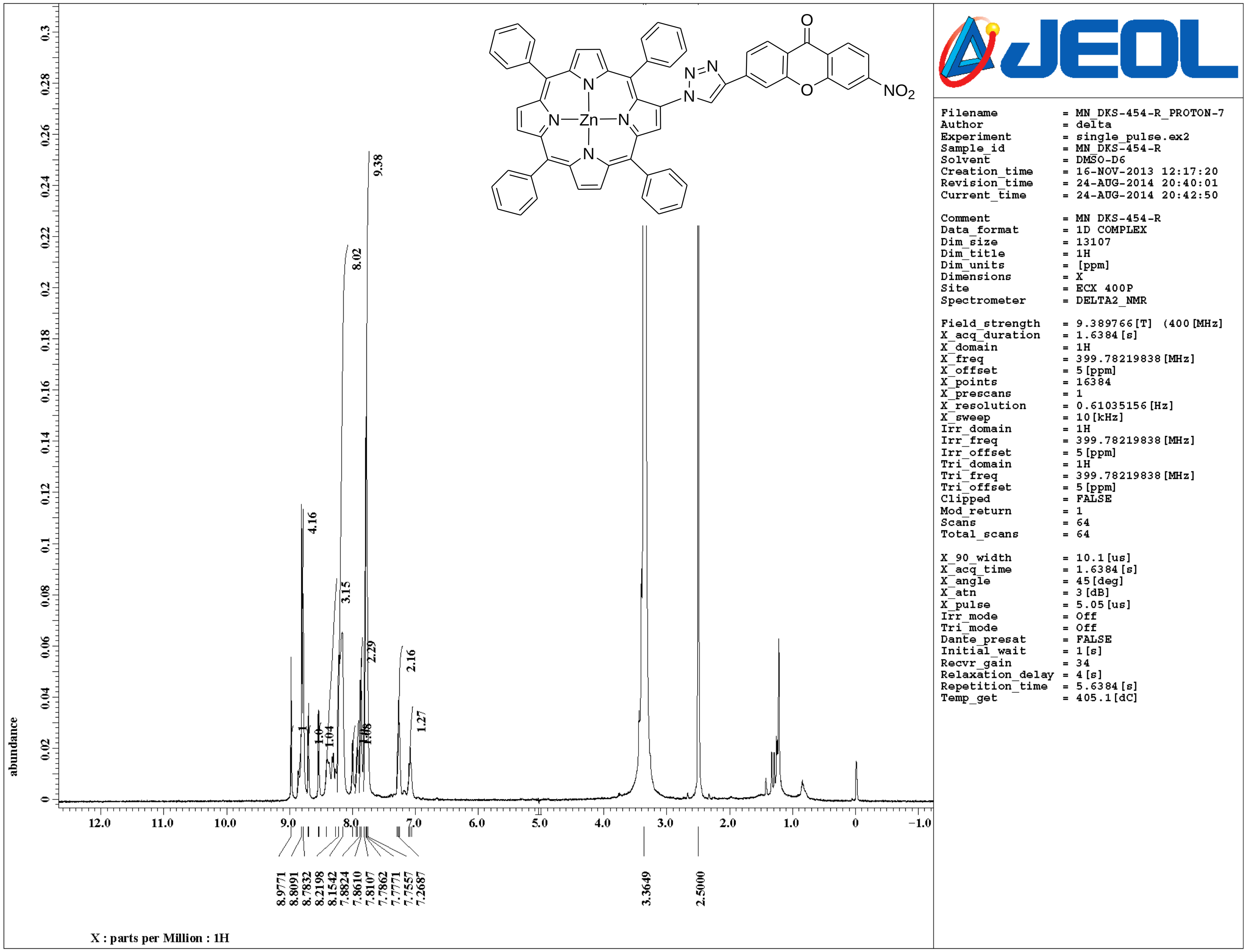

Figure S11: ^{1}H NMR spectrum of compound **6f** in DMSO-d_6.

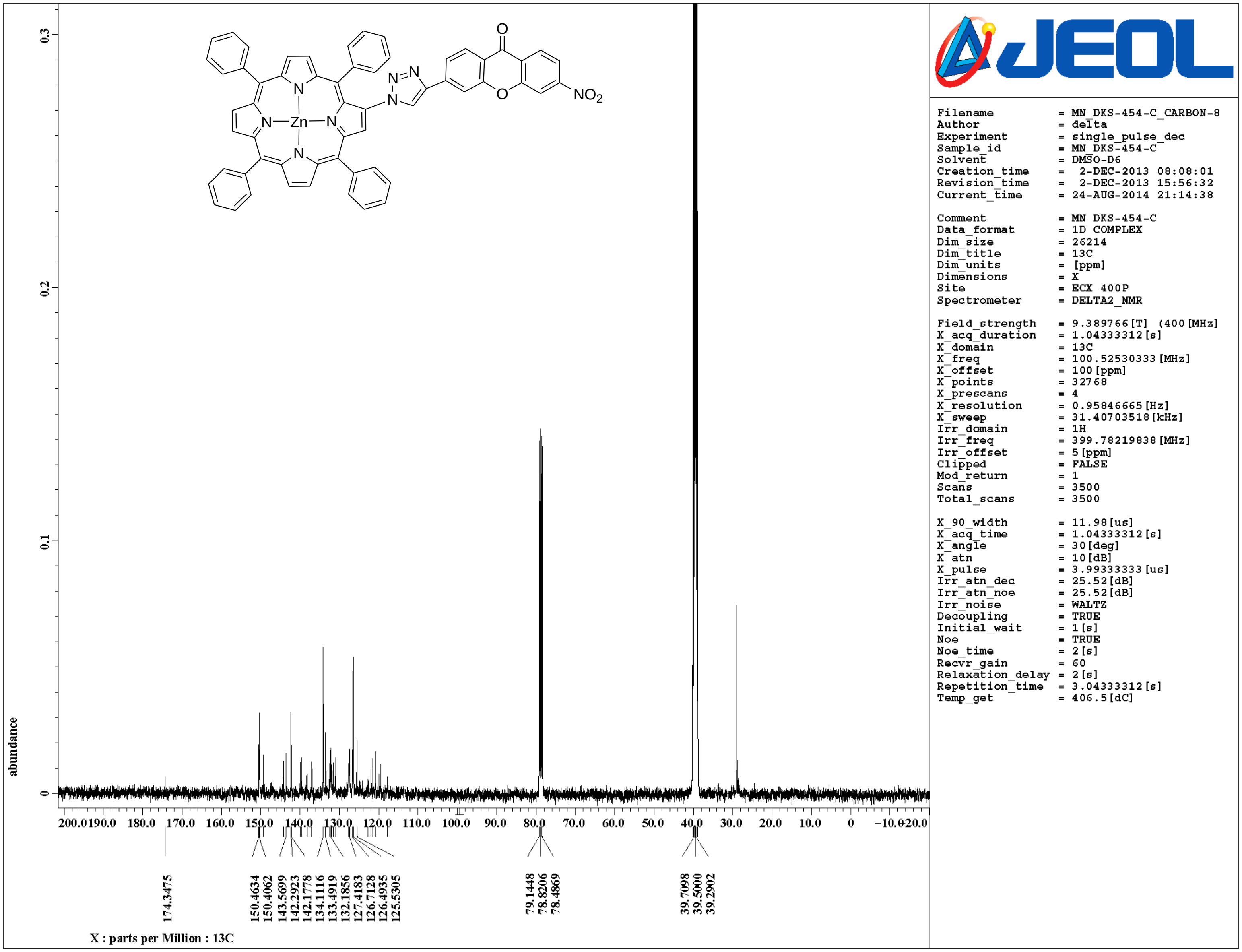

Figure S12: ^{13}C NMR spectrum of compound **6f** in $CDCl_3$ + DMSO-d_6.

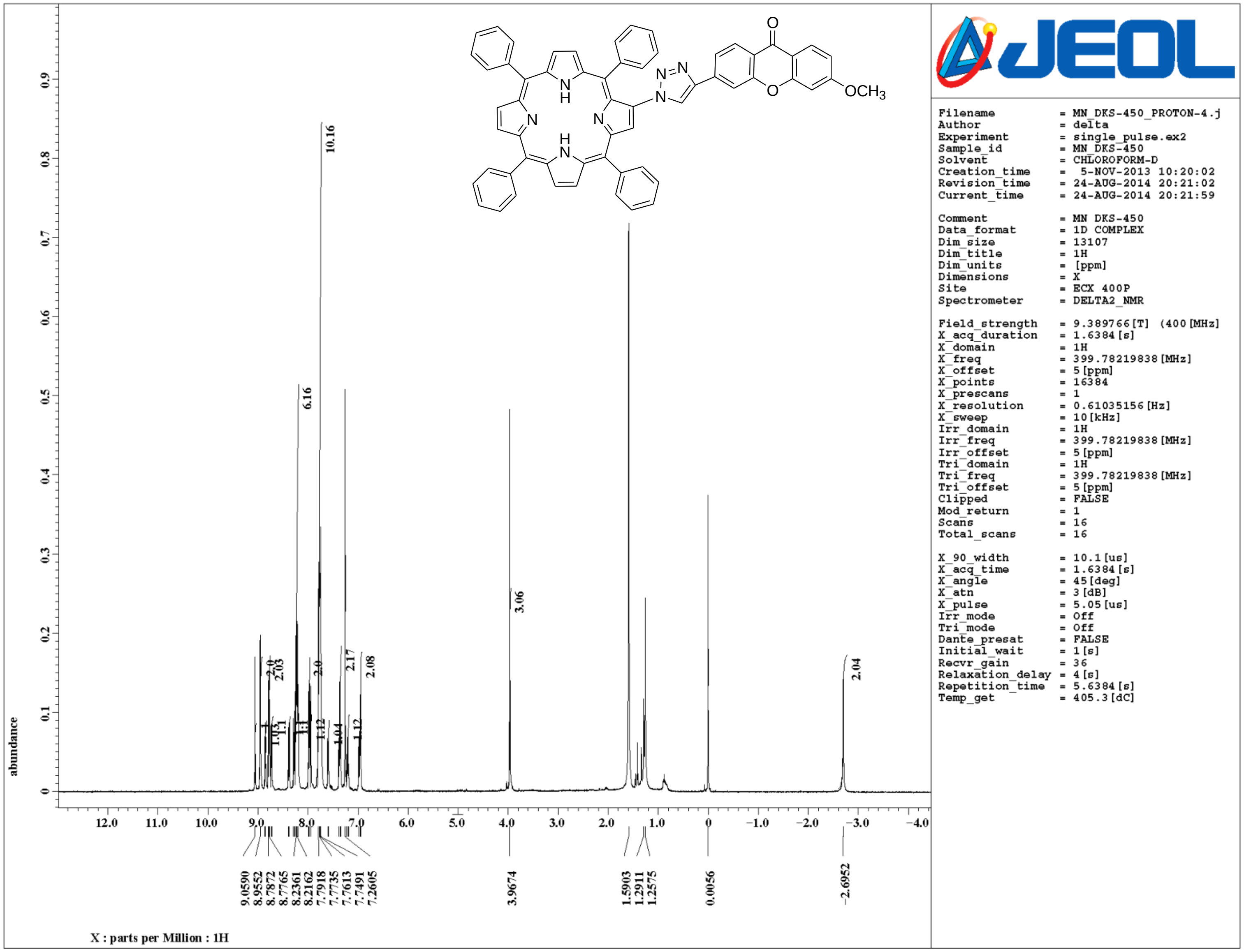

Figure S13: ^{1}H NMR spectrum of compound **6h** in $CDCl_3$.

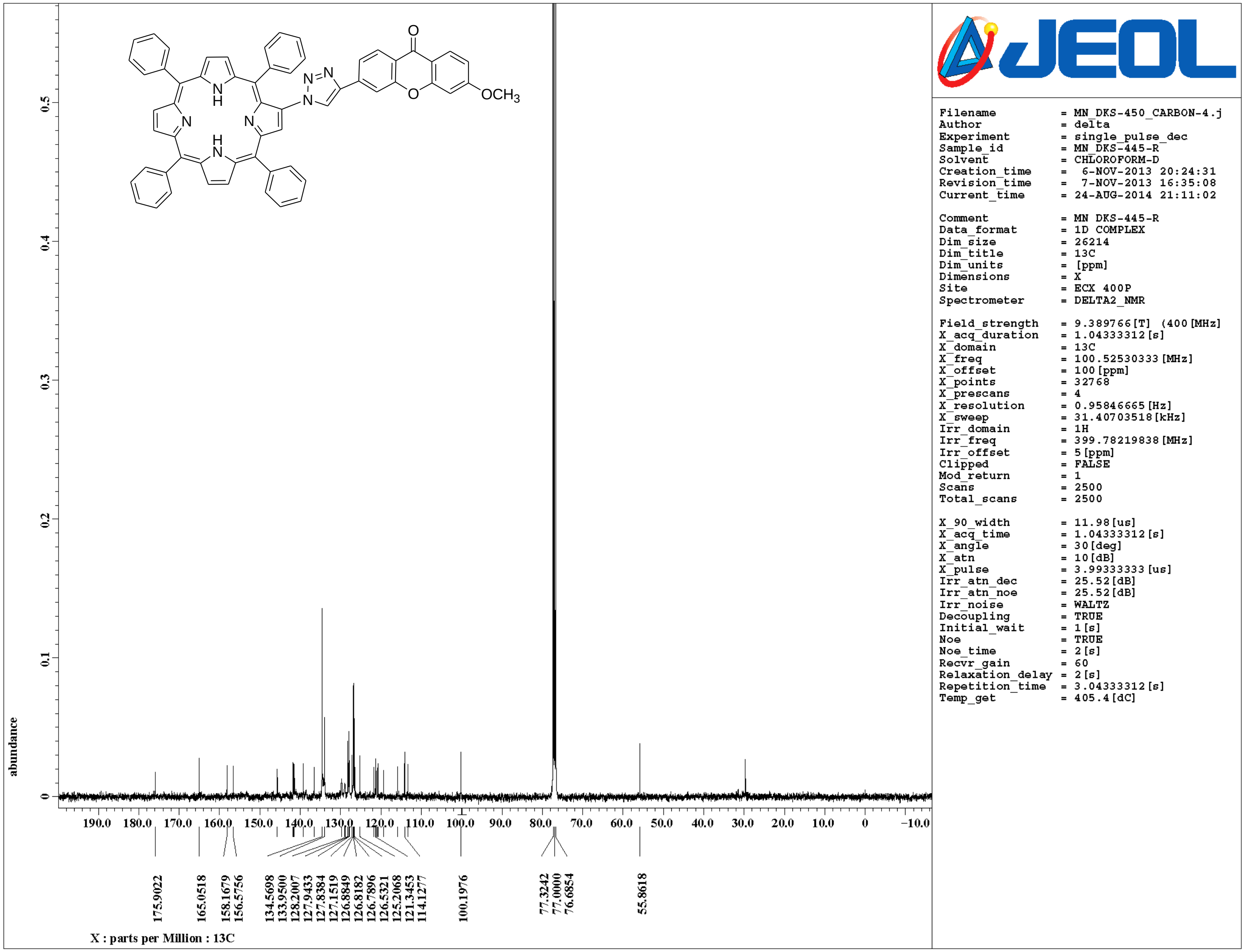

Figure S14: ^{13}C NMR spectrum of compound **6h** in $CDCl_3$.

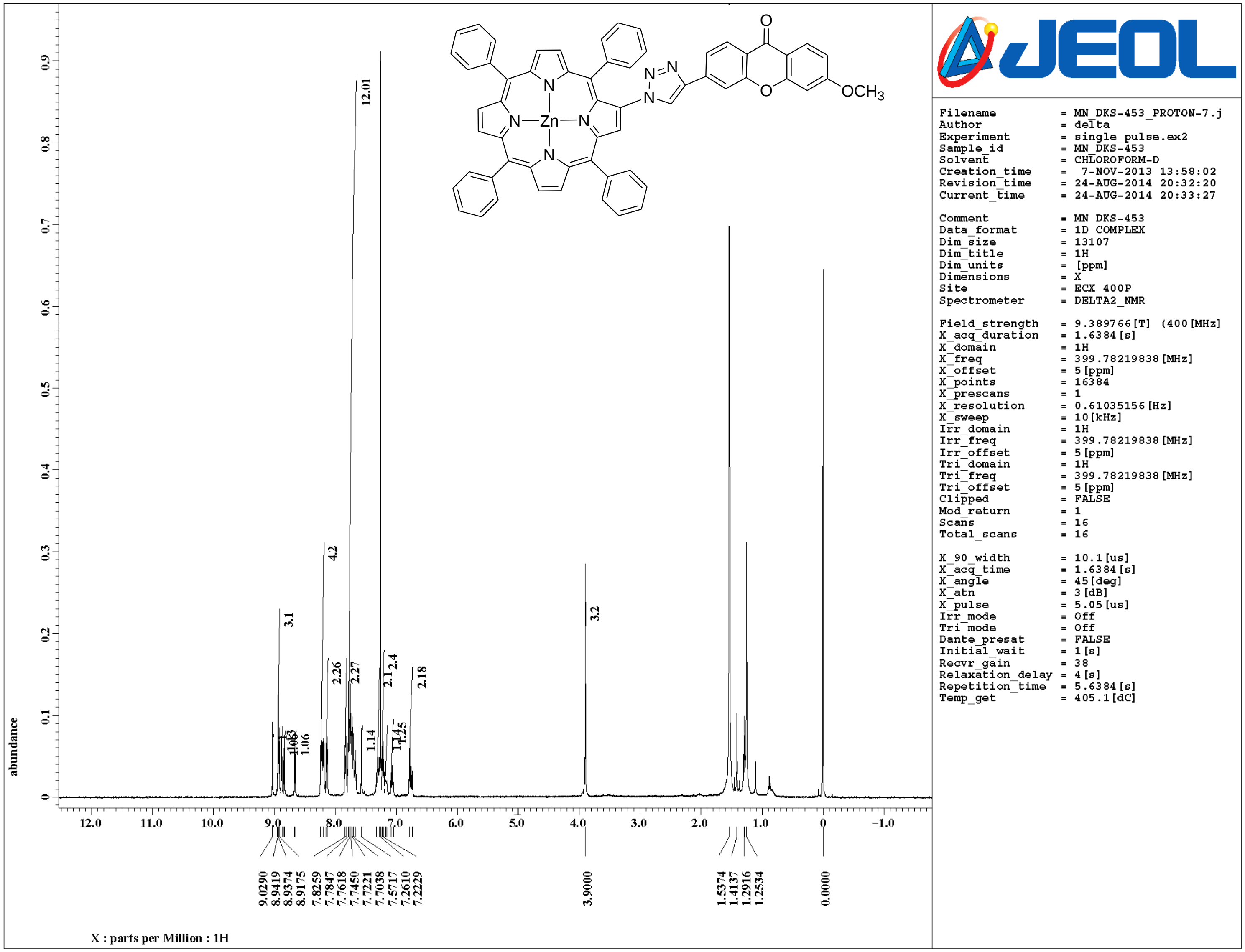

Figure S15: ^{1}H NMR spectrum of compound **6i** in $CDCl_3$.

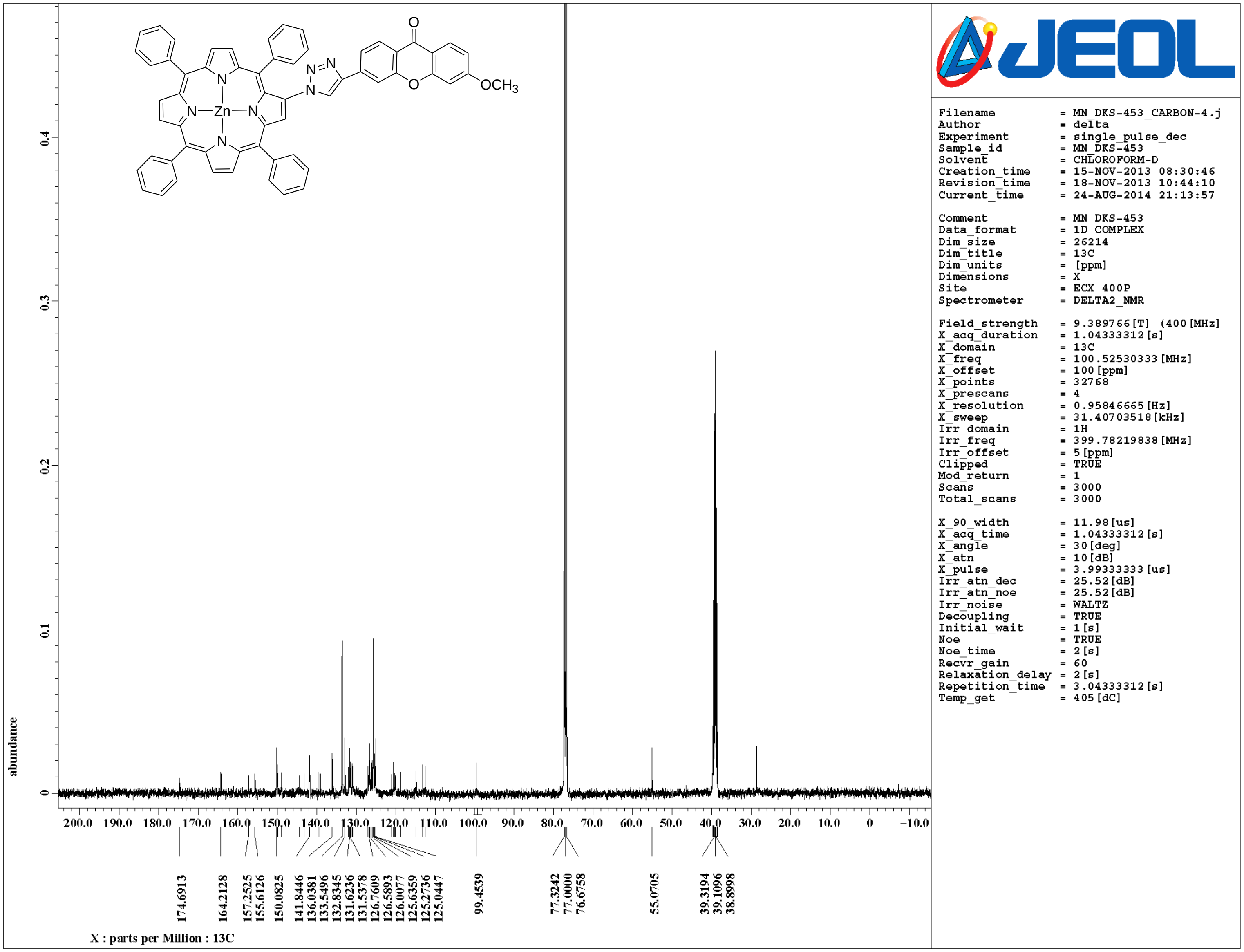

Figure S16: ^{13}C NMR spectrum of compound **6i** in $CDCl_3$ + DMSO-d_6.

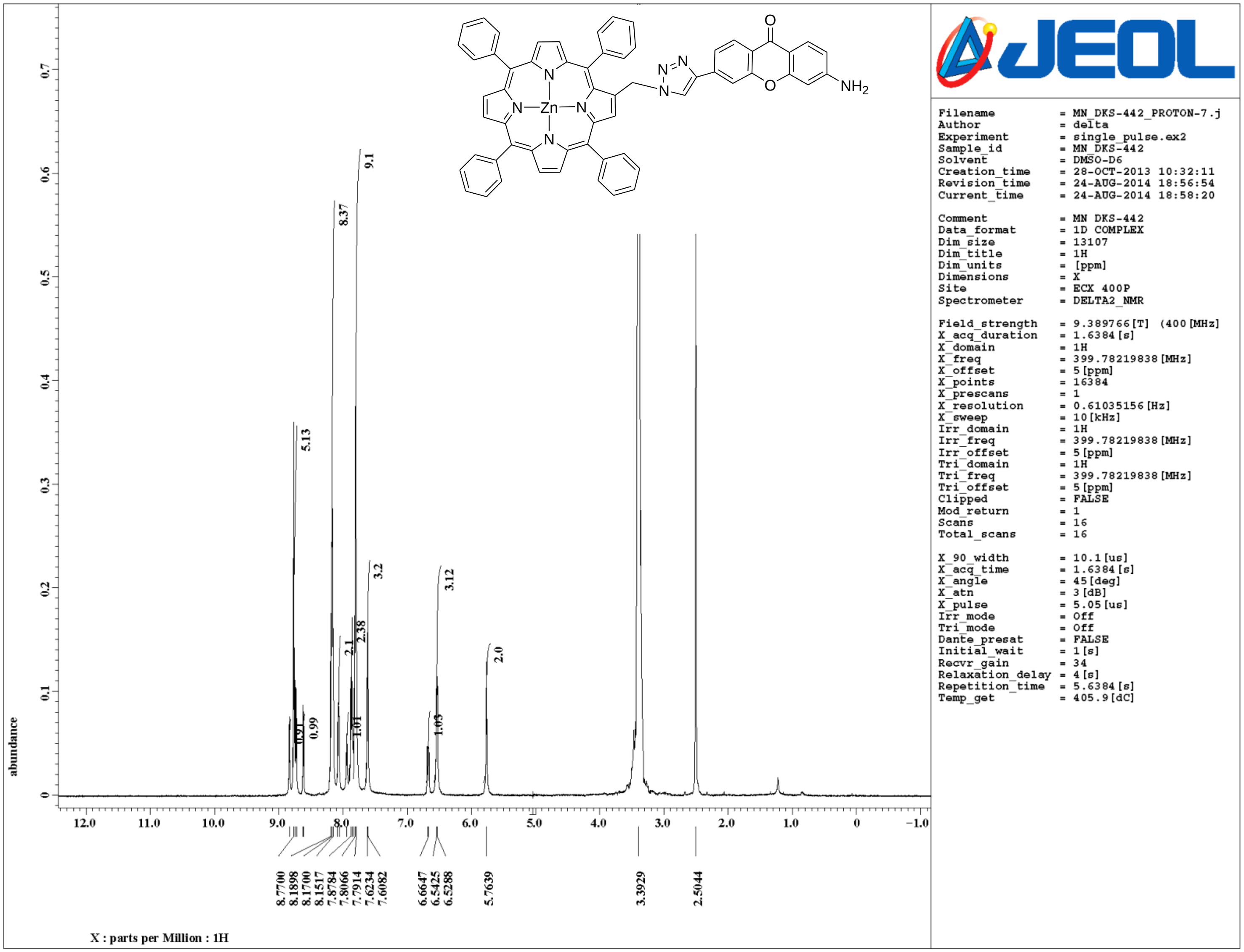

Figure S17: ^{1}H NMR spectrum of compound **7a** in DMSO-d_6.

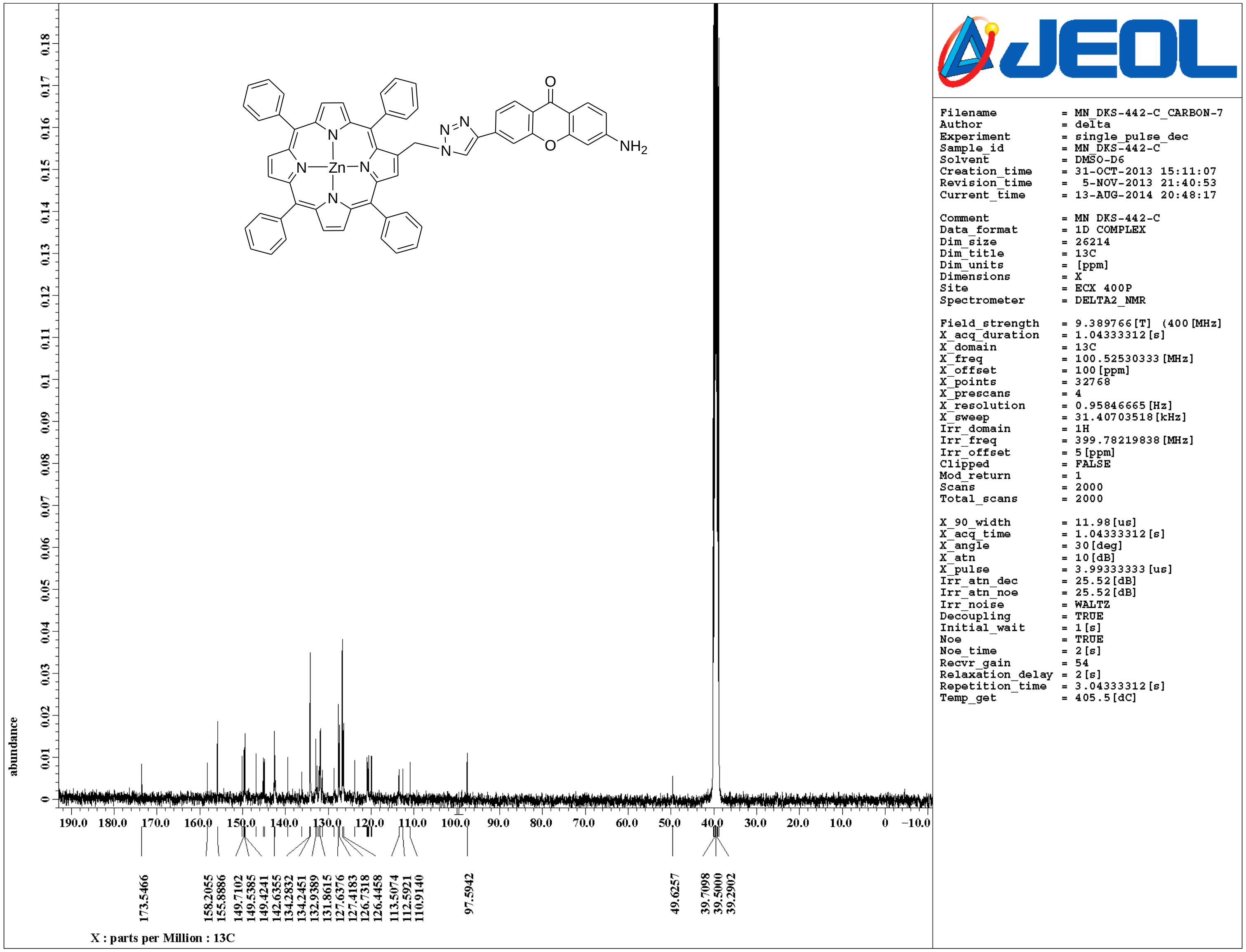

Figure S18: ^{13}C NMR spectrum of compound **7a** in DMSO-d_6.

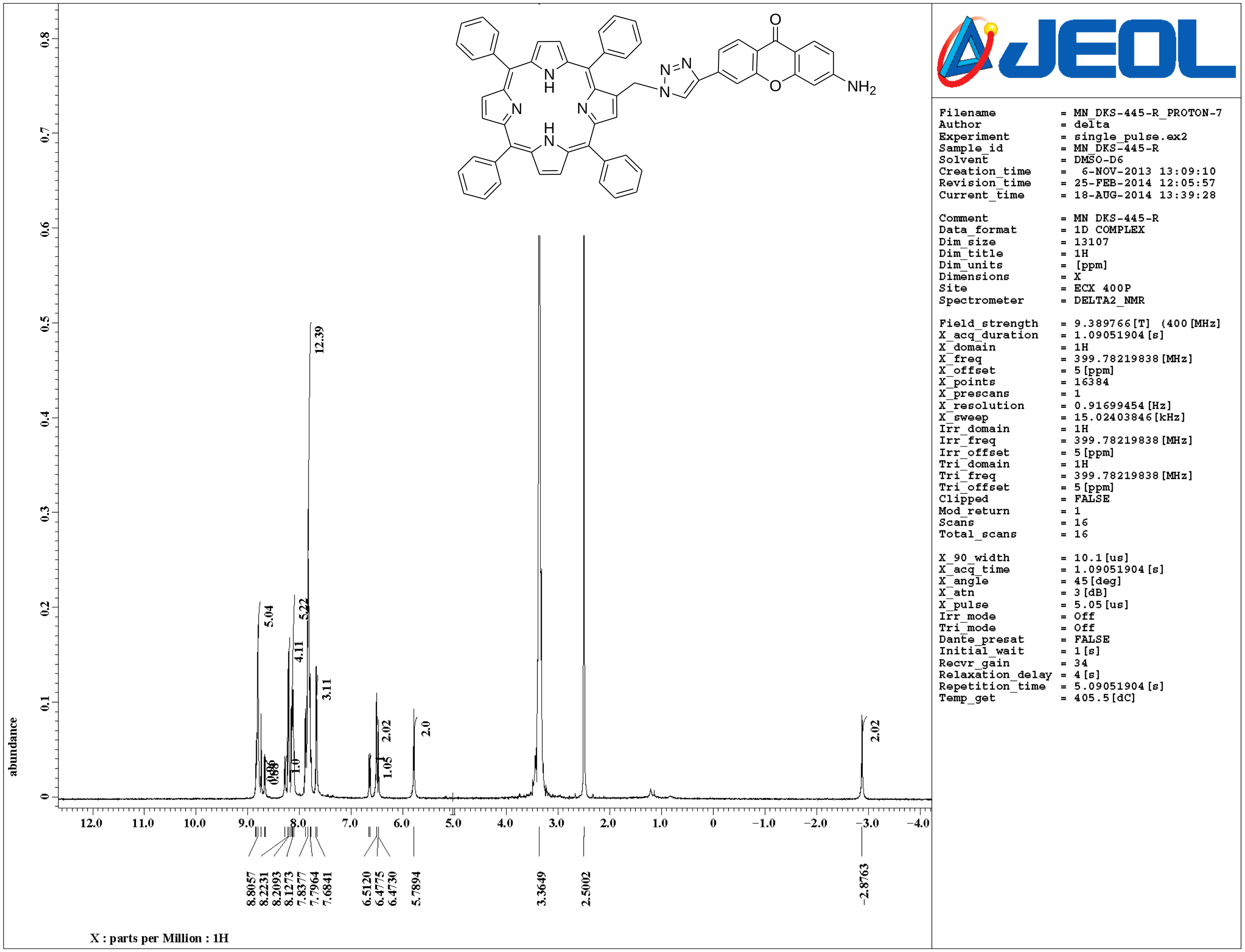

Figure S19: ^{1}H NMR spectrum of compound **7b** in DMSO-d_6.

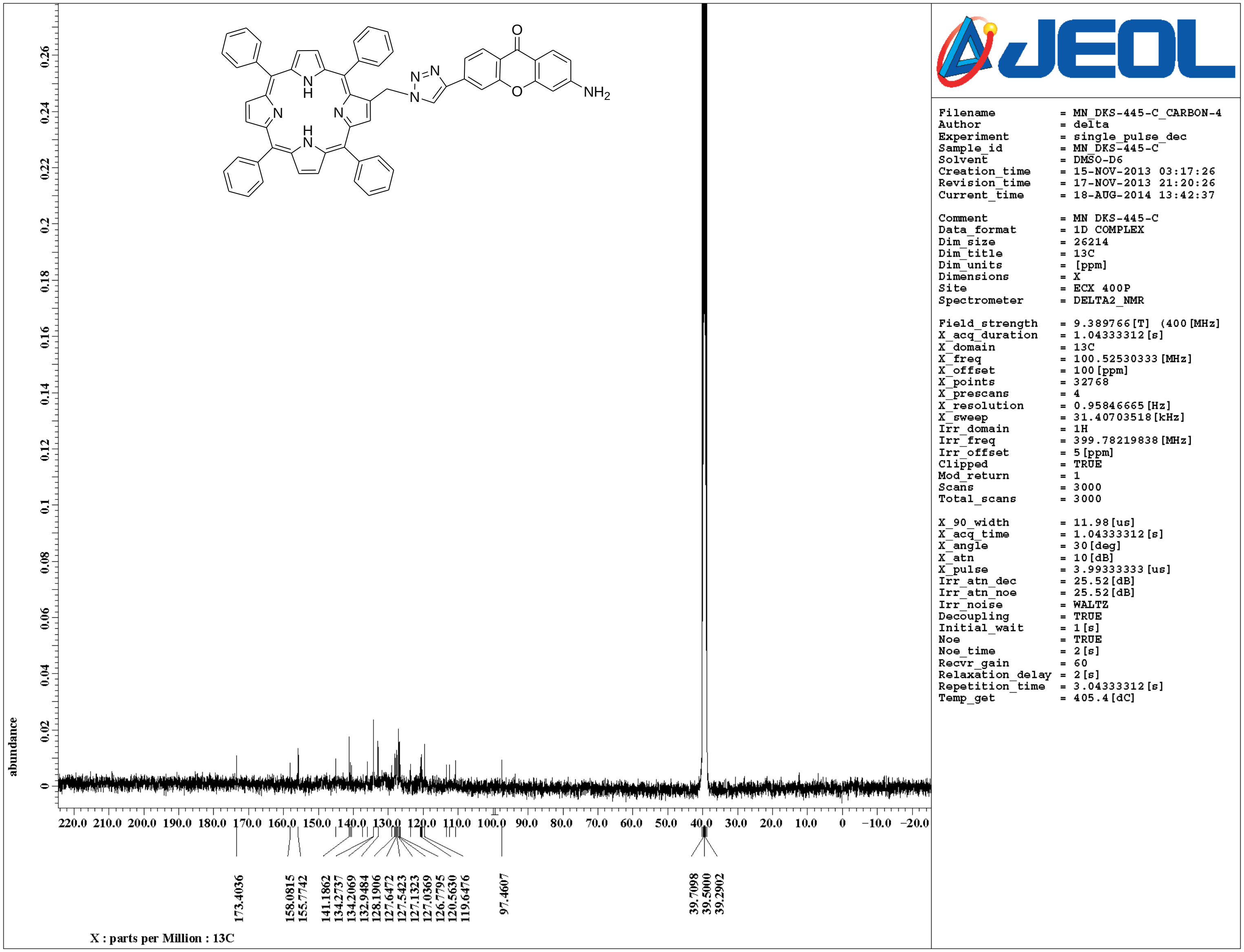

Figure S20: ^{13}C NMR spectrum of compound **7b** in DMSO-d_6.

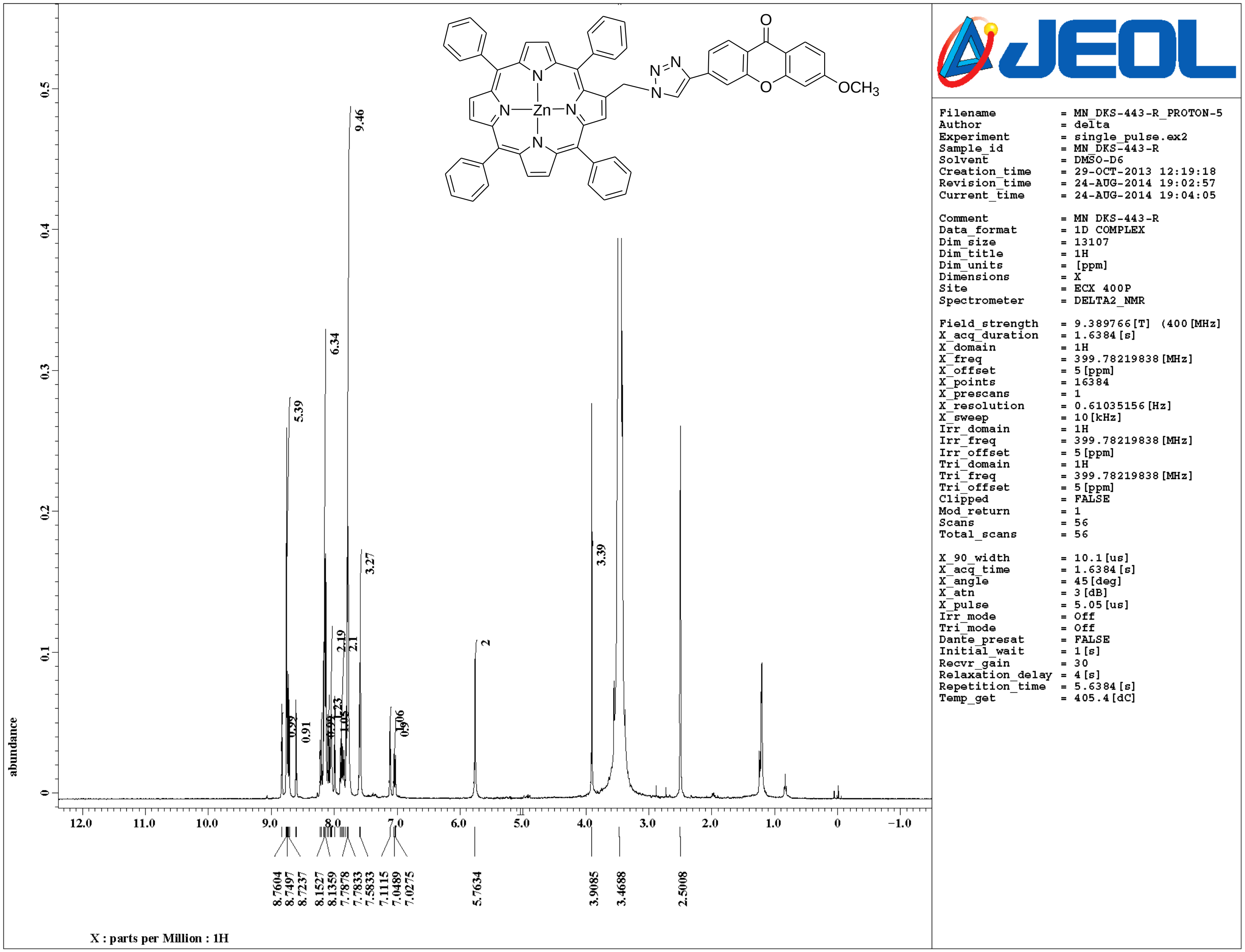

Figure S21: ^{1}H NMR spectrum of compound **7c** in DMSO-d_6.

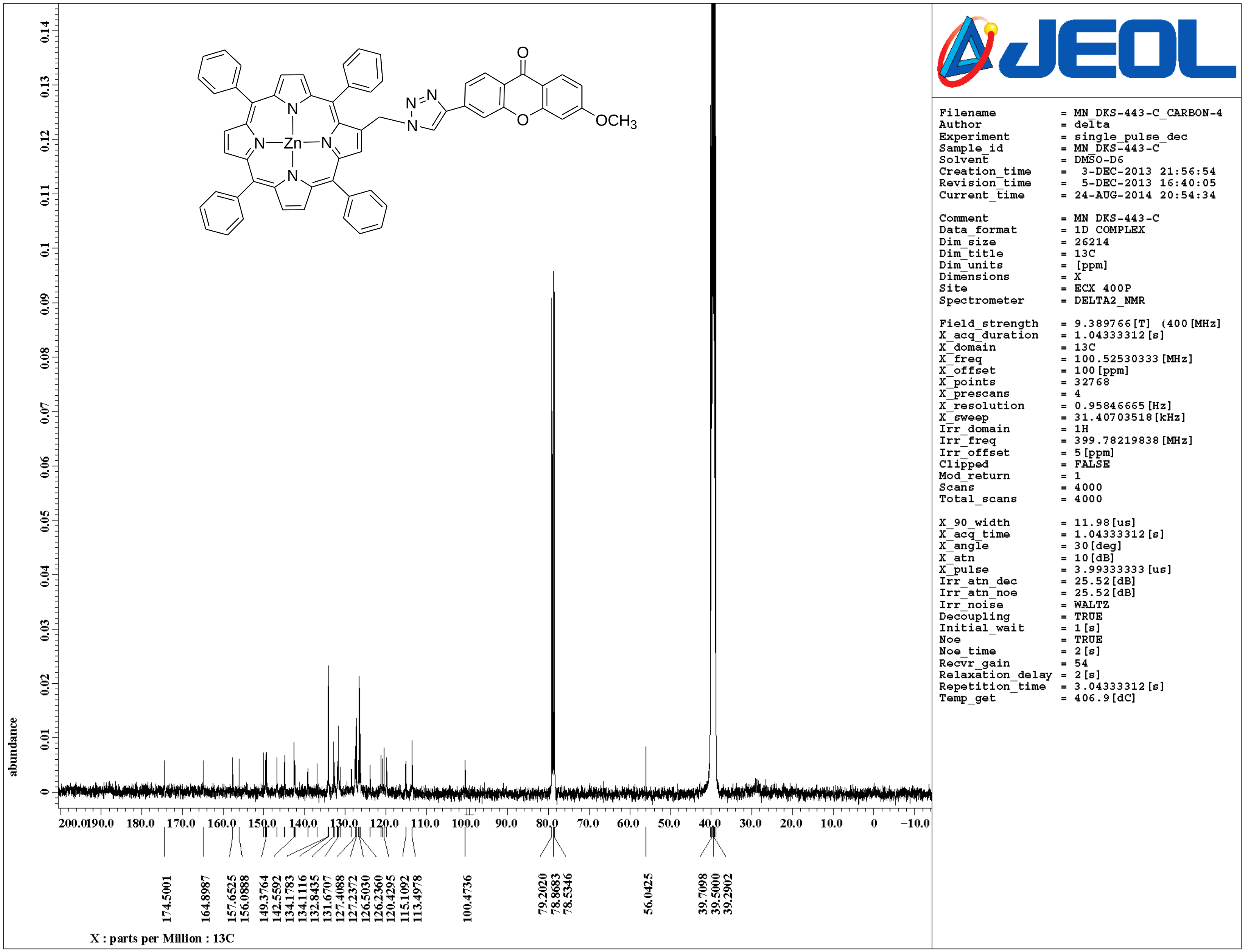

Figure S22: ^{13}C NMR spectrum of compound **7c** in $CDCl_3$ + DMSO-d_6.

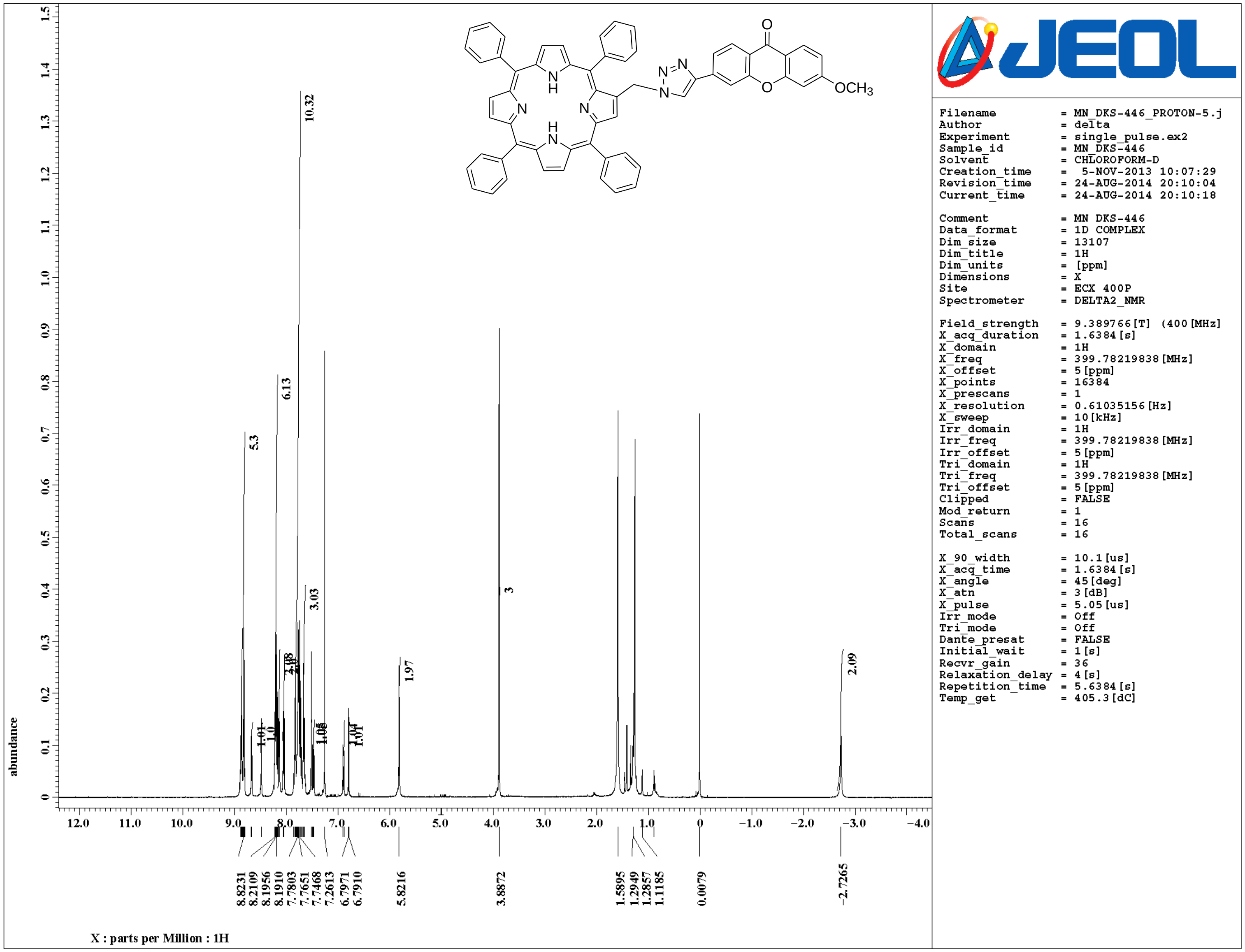

Figure S23: ^{1}H NMR spectrum of compound **7d** in $CDCl_3$.

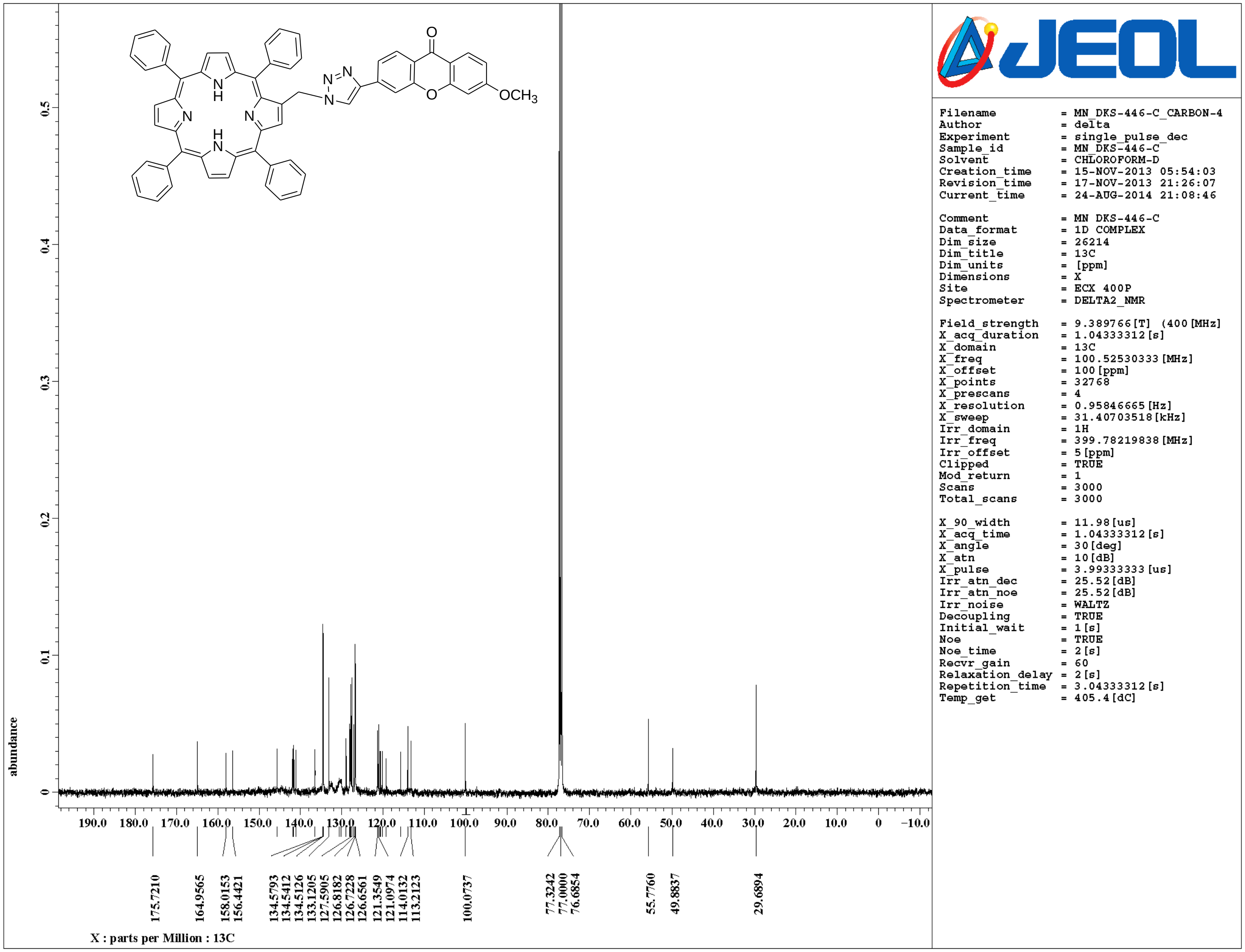

Figure S24: ^{13}C NMR spectrum of compound **7d** in $CDCl_3$.

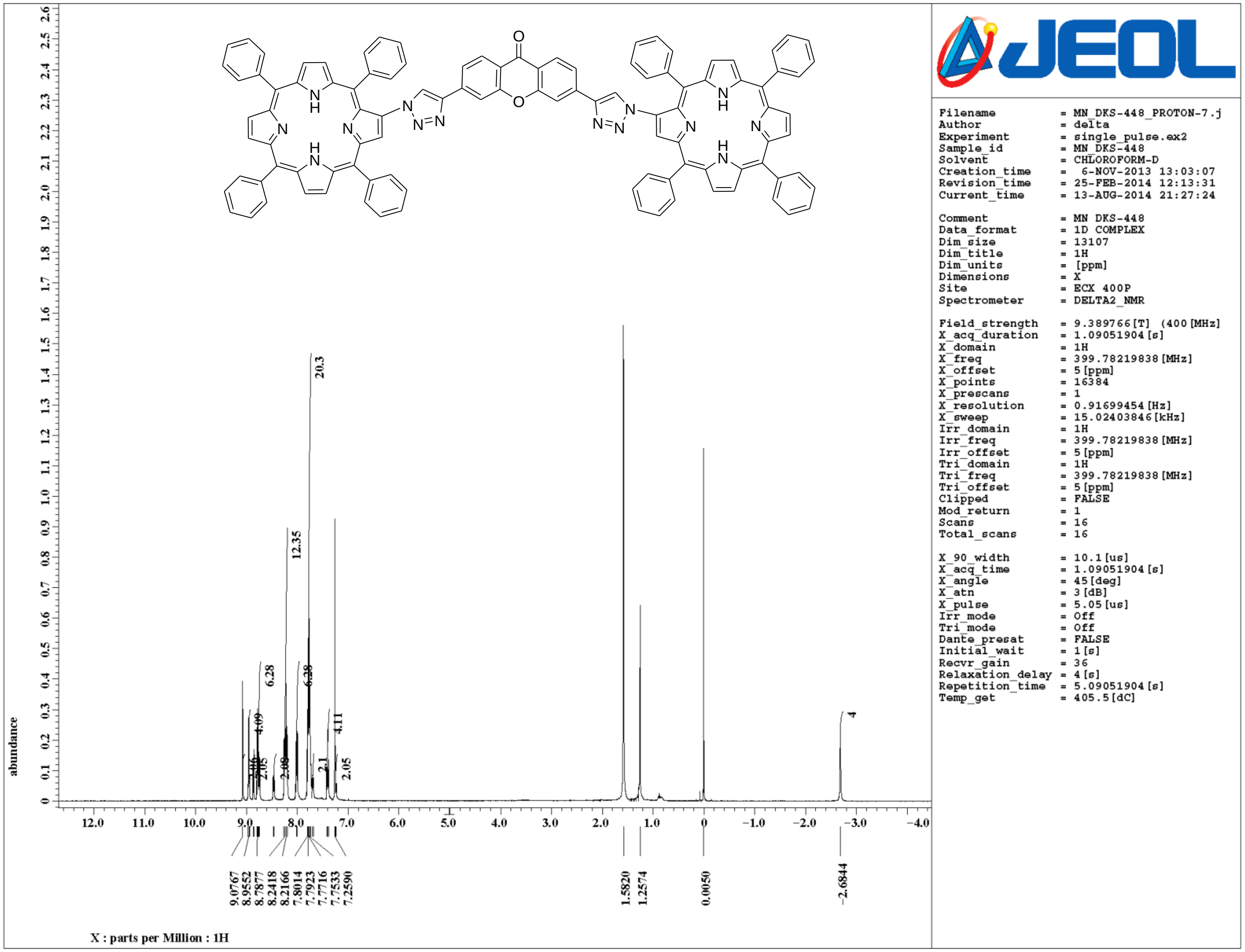

Figure S25: ^{1}H NMR spectrum of compound **12b** in $CDCl_3$.

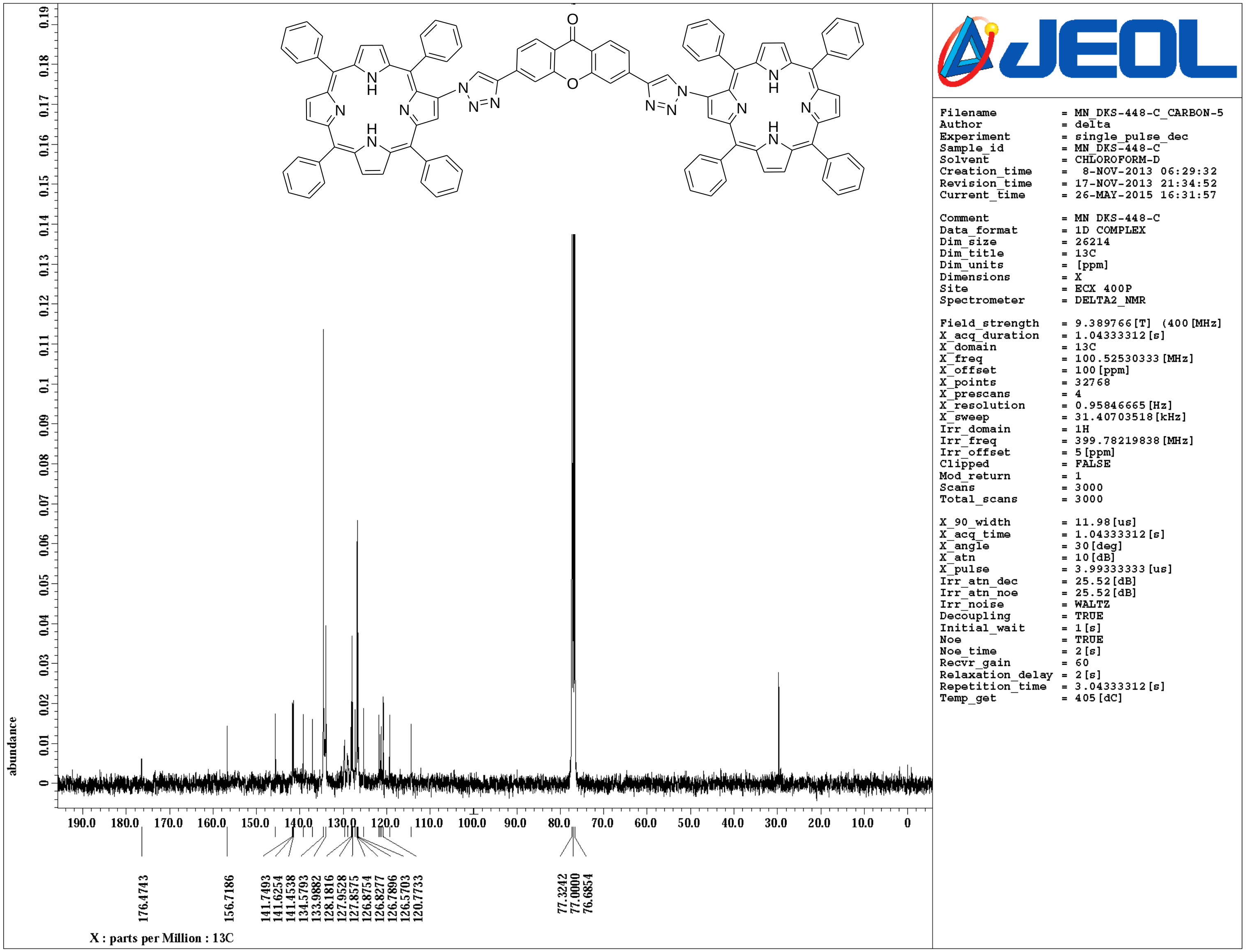

Figure S26: ^{13}C NMR spectrum of compound **12b** in $CDCl_3$.

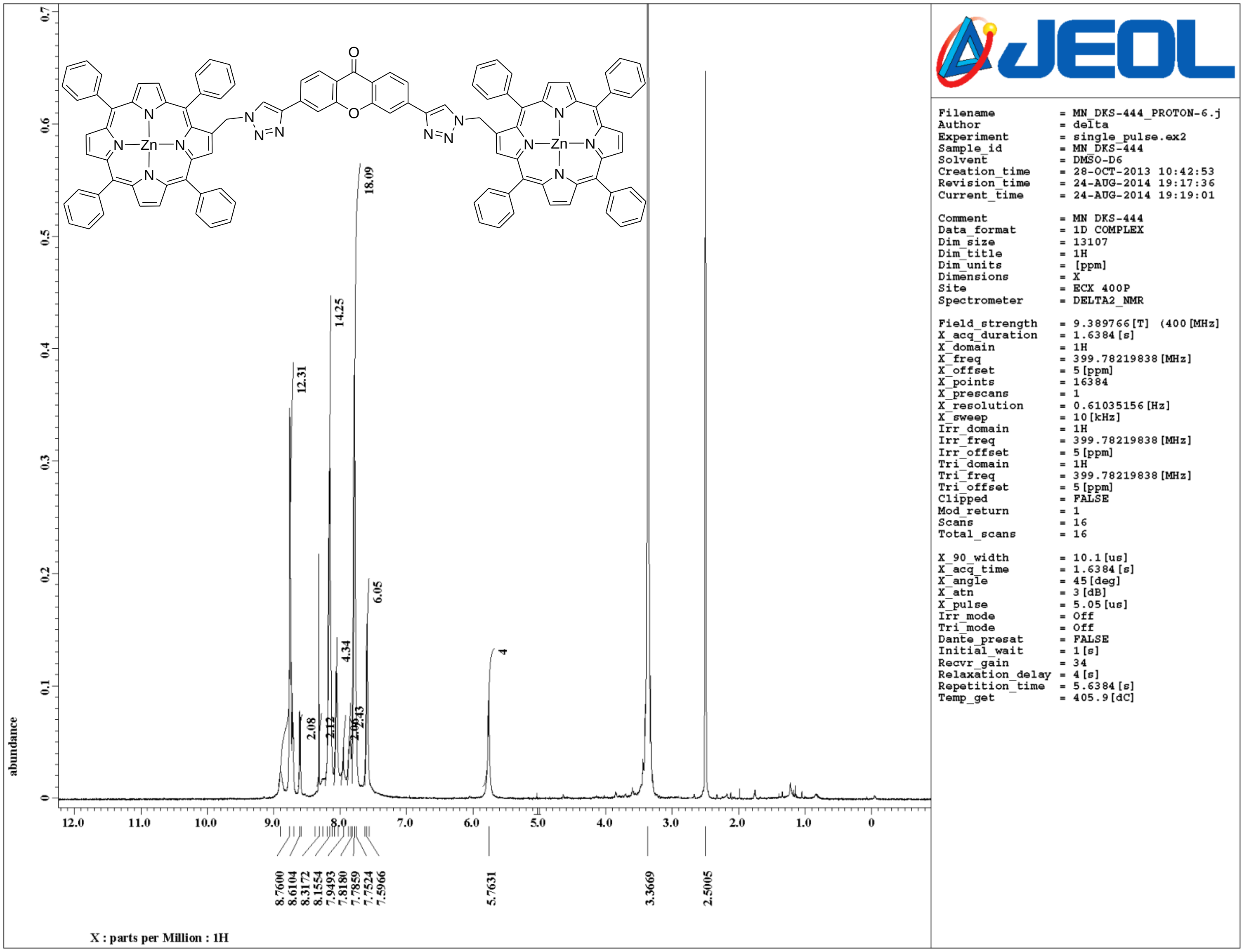

Figure S27: ^{1}H NMR spectrum of compound **13a** in DMSO-d_6.

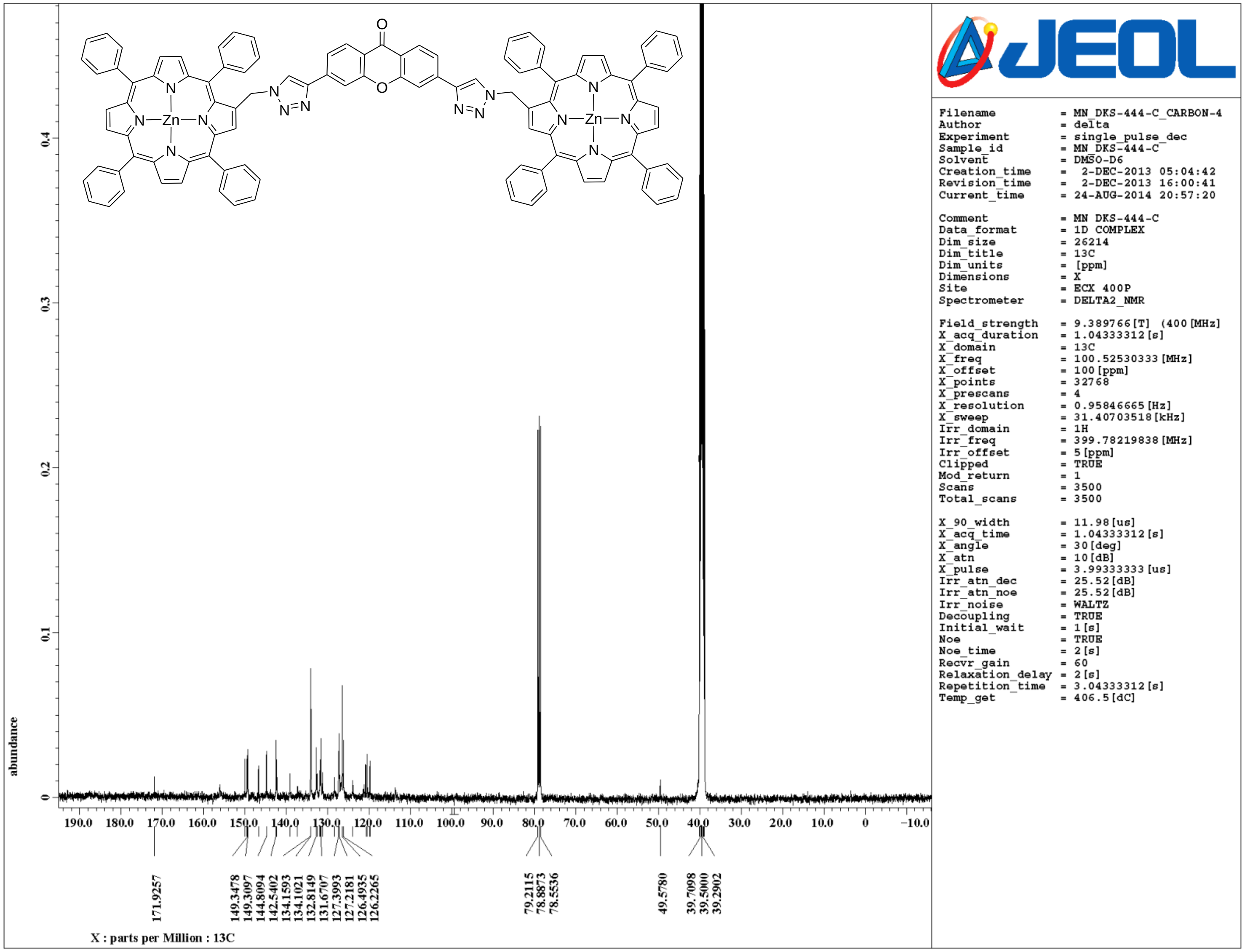

Figure S28: ^{13}C NMR spectrum of compound **13a** in $CDCl_3$ + DMSO-d_6.

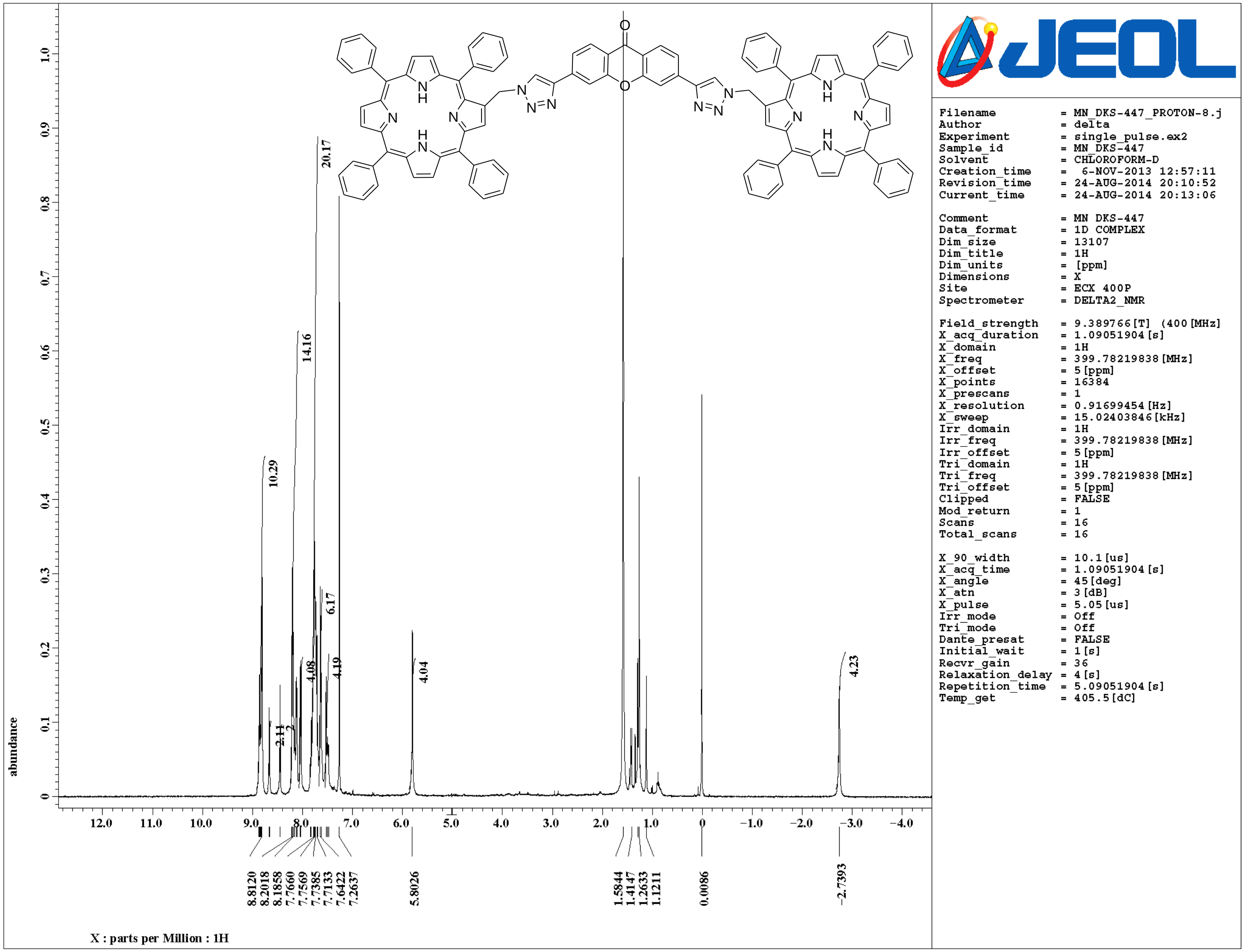

Figure S29: ^{1}H NMR spectrum of compound **13b** in $CDCl_3$.

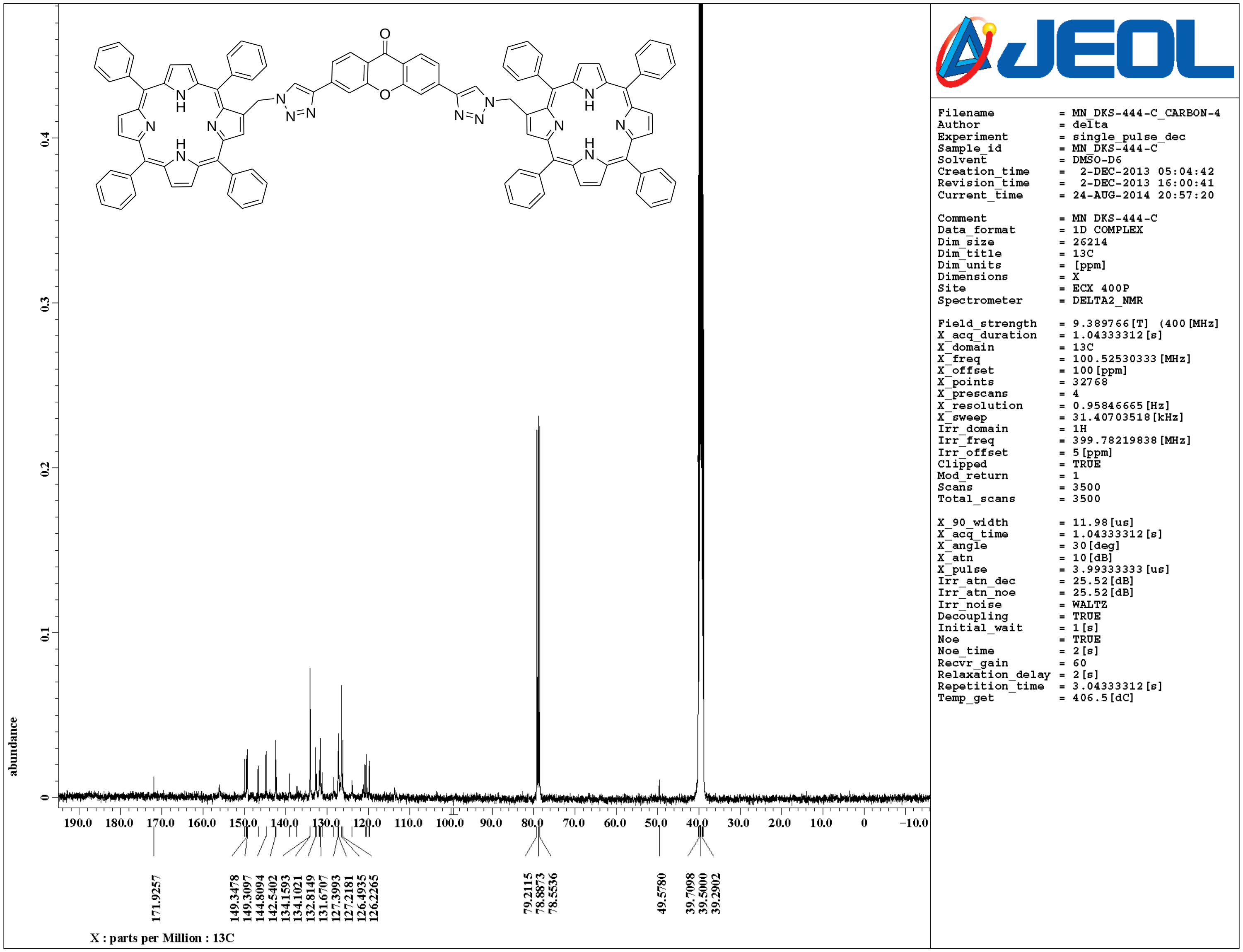

Figure S30: ^{13}C NMR spectrum of compound **13b** in $CDCl_3$ + DMSO-d_6.
